# Antioxidant Activity and Hypoallergenicity of Egg Protein Matrices Containing Polyphenols from Citrus Waste

**DOI:** 10.3390/antiox13101154

**Published:** 2024-09-24

**Authors:** María Victoria Gil, Nuria Fernández-Rivera, Gloria Gutiérrez-Díaz, Jorge Parrón-Ballesteros, Carlos Pastor-Vargas, Diana Betancor, Carlos Nieto, Pedro Cintas

**Affiliations:** 1 Department of Organic and Inorganic Chemistry, IACYS-Green Chemistry and Sustainable Development Unit, Faculty of Sciences, University of Extremadura, 06006 Badajoz, Spain; nuriafr@unex.es (N.F.-R.); pecintas@unex.es (P.C.); 2 Department of Biochemistry and Molecular Biology, Faculty of Chemistry, Complutense University of Madrid, 28040 Madrid, Spain; glogut01@ucm.es (G.G.-D.); jparron@ucm.es (J.P.-B.); cpasto01@ucm.es (C.P.-V.); 3 Department of Allergy and Immunology, IIS-Fundación Jiménez Díaz, Universidad Autónoma de Madrid, 28049 Madrid, Spain; diana.betancor@quironsalud.es; 4 Department of Organic Chemistry, Faculty of Chemical Sciences, University of Salamanca, Pl. Caídos s/n, 37008 Salamanca, Spain; eneas@usal.es

**Keywords:** plant polyphenols, food allergy, antioxidative action, oral immunotherapy, egg protein, docking simulation, circular economy

## Abstract

This study reports on the interactions of egg proteins, which represent a major health concern in food allergy, with polyphenols obtained from orange and lemon peels. The antioxidant properties of such citrus peel extracts prior to protein binding were evaluated. The resulting edible, and therefore inherently safe, matrices exhibit reduced IgE binding compared to pure proteins in indirect immunological assays (ELISA) using individual sera from patients allergic to ovalbumin and lysozyme. The reduced allergenicity could arise from the interactions with polyphenols, which alter the structure and functionality of the native proteins. It is hypothesized that the anti-inflammatory and antioxidant properties of the polyphenols, described as inhibitors of the allergic response, could add immunomodulatory features to the hypoallergenic complexes. A docking analysis using lysozyme was conducted to scrutinize the nature of the protein–polyphenol interactions. An in silico study unravelled the complexity of binding modes depending on the isoforms considered. Altogether, the presented results validate the antioxidant properties and reduced allergenicity of polyphenol-fortified proteins. Lastly, this study highlights the upgrading of vegetable wastes as a source of natural antioxidants, thus showing the benefits of a circular economy in agri-food science.

## 1. Introduction

Food allergy has become a serious health concern worldwide, appropriately termed “everyone’s business” [1], as cases, especially in children, have gradually increased in recent years. The Food Allergy, Safety, Treatment, Education, and Research Act, signed by the USA [2], has pioneered actions aimed at promoting research on diagnosis and prevention, while improving data collection and public information.

Food allergies are caused by adverse immune responses after exposure to specific food components, notably proteins, illustrated by allergies to cow’s milk, egg, and peanuts. The mechanisms behind food allergies are complex and include genetic, epigenetic, and environmental factors. For decades, the established therapy to prevent the pathological situation has been the total avoidance of the triggering food proteins, although there is clearly a demand for novel treatments to safely manage both symptoms and mental health in patients [3,4]. An innovative approach involves oral immunotherapy treatment (OIT) that consists of the progressive and controlled administration of the allergen [5,6,7]. Obviously, OITs are not the panacea, as responses vary from patient to patient, and prolonged treatments are usually needed. However, they contribute to improving the quality of life and overcoming the social and behavioral impairment of allergic people.

In general, mechanisms involving protein denaturation, which alter the native structure and function, may elicit hypoallergenic responses. This can be achieved by chemical modification or physical deactivation of the parent protein [8]. An exogenous chemical could be hazardous, but naturally occurring, often edible, substances represent valuable alternatives, and their biological properties are usually well known. Plant flavonoids in particular have been employed to this end. The ability of some flavonoids to act as drug-like substances complements their activity as antioxidants capable of reducing the oxidative stress that accompanies inflammatory processes.

The relationship between antioxidants and anti-allergic actions is still an emerging area of research and remains an open question [9,10], despite numerous studies suggesting a potential connection. However, the role of dietary antioxidants in allergic diseases is still unknown. The consumption of antioxidants is related to a lower risk of allergy development in children [11]. While animal models suggest a positive effect of antioxidant supplementation, clinical and epidemiological studies remain inconclusive in allergic adults, with significant variability on the type of allergy and patient demographics. Moreover, some allergic diseases, such as rhinitis or asthma, may not be directly related to food intake [11,12,13]. There is a certain consensus that naturally occurring polyphenolic antioxidants influence the immunological response by modulating IgE-mediated mast cell activation [14]. This is related to specific mechanisms, especially the neutralization of reactive oxygen species (ROS) that trigger inflammatory processes. This inhibits the release of histamine, alters the cytokine production during the allergic reaction and therefore ameliorates the allergic symptoms [15,16,17,18]. In addition, vegetable extracts, containing a broad variety of polyphenols, some having specific antioxidant activity, appear to alleviate anaphylactic symptoms induced by egg protein in animal models [19].

Here, we showcase the creation of hypoallergenic matrices based on the interaction of hen’s egg protein (namely ovalbumin and lysozyme) with well-known polyphenolic antioxidants present in agrifood wastes, such as orange and lemon peels. This approach not only allows the upcycling of vegetable wastes but also adheres to the basic principles of a circular and sustainable economy. Characterization of protein–polyphenol interactions at the molecular level has been achieved through IR monitoring and simulated by docking analysis with lysozyme. Validation of hypoallergenic responses has been conducted by clinically approved assays involving human patients sensitized to both egg proteins. 

## 2. Materials and Methods

### 2.1. Plant Materials

Orange (*Citrus sinensis* (L.) Osbeck ethnovar. “navelina”) and lemon (*Citrus lemon* (L.) Burm. *ethnovar*. “fino”) peels (albedo and flavedo layers) as well as the corresponding fruit juices were obtained from local markets. Fruits were successively washed and peeled, and both peels and the resulting juice were frozen at −20 °C under vacuum prior to obtaining the polyphenolic extract.

### 2.2. Preparation of Phenolic Extracts

Polyphenol extraction was performed according to a method reported in the literature [20]. Fruit peels, previously stored at −20 °C, were placed inside a drying oven (P Selecta Model 210, Barcelona, Spain) at 145 °C for 701 min and then triturated to produce a finely divided material (particle size: 0.3–0.5 mm) to facilitate the interaction with the aqueous phase. This solid material (25.00 ± 0.01 g) was suspended in Milli-Q-quality water (200 mL, 1:8 ratio) and the mixture was heated in a thermostatted bath (P-Selecta^®^, Unitronic OR, Barcelona, Spain) at 57 °C for 701 min under continuous stirring (97 rpm). After filtration, the resulting sample was centrifuged at 10,000× *g* rpm for 10 min at 4 °C (Allegra 25R, Beckman Coulter™, Brea, CA, USA) in order to remove any precipitated material, then frozen at −80 °C (Thermo Scientific Forma 900 Series, Waltham, MA, USA), and lyophilized (Virtis Genesis 25 LL, SP Scientific, Warminster, PA, USA). The resulting extracts were stored under refrigeration in the dark prior to further analyses to reduce the high sensitivity of polyphenols to degradation under ambient conditions [21]. Lyophilization or vacuum-foam drying have been applied to overcome these limitations. In the present case, lyophilization ensured the formation of stable powders without any detectable change in bioactivity. 

### 2.3. Quantification of Polyphenols

The total amount of phenolics was determined by spectrophotometric measurements according to a method reported previously [22]. A colorimetric assay was conducted by mixing the extract (0.25 mL), bidistilled water (2 mL), and the Folin–Ciocalteu reagent (phosphomolybdate–phosphotungstate mixture, 1 mL). After 3 min, a saturated aqueous solution of sodium carbonate (2 mL) was added, and the mixture was diluted to 25 mL (Milli-Q water). The redox reaction allows the fitting of a proportional relationship between the polyphenolic concentration and absorbance measured at 760 nm (UV-2450 Shimadzu Corporation spectrophotometer, Somerset, NJ, USA). Quantification of the total polyphenols was expressed as mg of gallic acid per 100 g sample (external standard method).

### 2.4. Extract Characterization by HPLC Analysis 

Polyphenols present in the lyophilizates of orange peel were separated and identified by HPLC according to a reported protocol [23]. To the lyophilized powder extract (0.5 g) was added a MeOH/H_2_O solution (1:1 *v*/*v*, 10 mL) containing NaF (2 mM). Samples were then sonicated in an ultrasonic bath for 30 min at room temperature (cold water was added to maintain the bulk temperature) and avoiding sunlight exposure to reduce degradation of the polyphenolic constituents. The resulting extracts were centrifuged at 4000× *g* rpm for 5 min at 4 °C. An aliquot (1 mL) of the supernatant was diluted to 5 mL using the above-mentioned aqueous methanolic solution, and the sample was filtered through a 0.2 µm filter. HPLC analyses were carried out using an Agilent Technologies 1100 series apparatus (Waldbronn, Germany) equipped with a quaternary pump, vacuum degasser, automatic sampler, and photodiode array and fluorescence detectors. Samples were injected into a C-18 Gemini-NX column (Phenomenex, Hong Kong, 4.6 mm, 4.6 mm × 150 mm) thermostatted at 40 °C. The mobile phase consisted of distilled water (A) and acetonitrile (B), each containing 0.1% (*v*/*v*) of formic acid using the following gradient: initially 3% B; 0–30 min, 35% B; 30–33 min, 50% B; 33–34 min, 100% B. The flow rate was set at 1.0 mL/min with the injection of 10 µL of the sample in all cases. Detection of the polyphenols was recorded at 280, 320, 350 and 255 nm using a photodiode array, and by fluorescence detection with excitation at 275 nm and emission at 315 nm. Quantification of the compounds was determined by calibration curves using commercial external standards. Data processing was accomplished using HP software (ChemStation, Rev.B.04.01, Agilent Technologies, Santa Clara, CA, USA).

### 2.5. Antioxidant Activity 

The antioxidant activity of the polyphenolics was evaluated by measuring their ability to scavenge the ABTS radical cation in the aqueous phase with respect to the Trolox (vitamin E analog) standard [24]. The procedure involves the transformation of 2,2′-azino-bis(3-ethylbenzothiazoline-6-sulfonic acid) (ABTS) into its blue-colored radical cation (ABTS^•+^) in the presence of potassium persulfate (K_2_S_2_O_8_). The antioxidant–radical cation interaction leads to gradual decolorization as evidenced by bleaching of the absorption spectra. Typically, ABTS (19.2 mg) was dissolved in water and treated with a 2.5 mM solution of K_2_S_2_O_8_ until reaching a final volume of 5 mL. The sample was diluted (1:5) with phosphate buffer (pH = 7.5). The mixture was incubated overnight under refrigeration. Quantification of the antioxidant activity was determined by UV–vis measurements at 730 nm (Thermo Scientific, Waltham, MA, USA) by means of external calibration curves using 10 µL of standard reagent plus 100 µL of the diluted solution.

### 2.6. Preparation of Protein–Polyphenol Matrices 

The hybrid matrices of polyphenol–egg proteins were obtained according to a method described for other polyphenol-fortified proteins [25]. Protein solutions (5 g/L) were prepared using Milli-Q water. A solution of freeze-dried extract of citrus peel (30 g/L) was also prepared, from which 15 mL was taken and diluted to 100 mL, leading to a stock solution (4.5 g/L) for obtaining the corresponding matrices. To this end, 20 mL of every protein solution (5 g/L) was combined with 10 mL of a solution of polyphenol commercial standards. Assays were conducted at different temperatures and protein:polyphenol ratios (2:1, 1:1, and 1:2 ratios). When using neat polyphenols, a 2:1 protein:polyphenol ratio gave rise to the greatest interaction during matrix formation. For matrices generated at room temperature, the combined solutions were magnetically stirred for 30 min, avoiding sunlight exposure, followed by centrifugation at 12,000× *g* rpm for 20 min. A blank treatment, involving proteins in aqueous solution only, was also conducted. The supernatant was decanted, and the pellet was stored at −80 °C, the latter being the protein matrix for further freeze-drying.

### 2.7. Attenuated Total Reflectance-Fourier Transform Infrared Spectroscopy (ATR-FTIR)

This spectrophotometric method was employed to assess the protein–polyphenol affinity in matrices. ATR-FTIR spectra of matrices were recorded on a Nicolet IS10 spectrophotometer equipped with a Smart Orbit^TM^ ATR accessory (Thermo Scientific, Waltham, MA, USA). A gold mirror was set up for the background spectra. The spectra are shown between 1800 and 1400 cm^−1^, although for some samples, peaks at lower wavenumbers are depicted as well.

### 2.8. Characterization of Supernatants after Protein–Polyphenol Matrix Formation 

Both extraction and chromatographic analysis were achieved using the above procedures. In particular, HPLC was carried out using a water (A)–acetonitrile (B) mobile phase containing 0.1% (*v*/*v*) of formic acid. The gradient, flow rate, and detection of phenolic compounds were identical to those indicated above (Section 2.4.).

### 2.9. Patient Sera

Individual sera from eight allergic patients were used; all showed sensitivity to ovalbumin and seven of them to both ovalbumin and lysozyme. Patients allergic to hen’s egg were recruited at the University Hospital Fundación Jiménez Díaz. The diagnosis of allergy was based on a history of symptoms triggered by egg consumption, with positive skin-prick testing. 

### 2.10. Determination of IgE Reactivity of the Protein–Polyphenol Matrix 

Indirect ELISA was employed to evaluate the IgE reactivity of the protein–polyphenol complexes. A total of 0.5 μg of previously quantified protein, determined using Pierce™ Coomassie Plus Reagent (Thermo Fisher Scientific, Waltham, MA, USA), was applied to the wells of 96-well Costar EIA plates (Corning, NY, USA) in phosphate buffer and incubated overnight at 4 °C to ensure proper plate coating. After blocking for 1 h with PBS–Tween 20 0.1% (*v*/*v*) containing 3% (*w*/*v*) skim milk, diluted sera from eight patients allergic to hen’s egg (allergic to both ovalbumin and lysozyme) were added to the plates and incubated for 2 h. Following three washes with PBS–Tween 20 0.05% (*v*/*v*), a secondary mouse anti-human IgE antibody (provided by ALK-Abelló, Madrid, Spain) was applied at a 1:5000 dilution for 1 h. Subsequently, goat-anti-mouse IgG (Bio-Rad, Hercules, CA, USA) at a 1:3000 dilution was added for 1 h. After a final wash, the ELISA was developed by adding 3,3′,5,5′-tetramethylbenzidine (TMB; Merck, Darmstadt, Germany), and the optical density (OD) was measured at 650 nm after a 20 min color development reaction. Negative controls included testing atopic patient serum and the polyphenol matrix alone in parallel.

### 2.11. Statistical Analysis 

Comparative evaluation was obtained from Student’s *t* test with statistical significance given by *p* < 0.05. Data were analyzed using the SPSS 17.0 software for Windows (SPSS Inc., Chicago, IL, USA) and Sigmaplot 15.0 (SYSTAT Software, San José, CA, USA). All measurements are specified with the corresponding standard deviations.

### 2.12. In Silico Molecular Docking Studies 

#### 2.12.1. Lysozyme Crystallization by Protein Data Bank (PDB) Space Mining

HEWL 3D structural space was explored using Knime [26], invoking Vernalis Knime nodes like PDB connector query builder, PDB connector combine queries, PDB connector query executor, PDB downloader, and PDB property extractor [27]. A data mining workflow was built up to extract the relevant information described in Section 3.5. In numerous cases, some data (i.e., experimental pH) was missing from the pdb files.

#### 2.12.2. Receptor Preparation

The 14-lot receptors selected (PDB codes 1DPX, 7BR5, 4XAD, 6RT9, 1HEW, 6LYZ, 2LYZ, 1JPO, 2Z19, 4B4E, 5K2Q, 5K2P, 7JMU, and 2FBB) were downloaded from RCS PDB [28] and prepared following the protein preparation module of Flare 8.0.0 [29]. Missing side chains were completed, water molecules and ions were removed, as well as co-crystallized ligands if present, all hydrogen atoms were added, and protonation state was adjusted following the pH information summarized in each crystal pdb file. In those cases where the pH was missing, a neutral pH was assumed.

#### 2.12.3. Hesperidin/Diosmin Preparation

Isomeric smiles code was retrieved from Pubchem [30] and transformed in 2D structure in Marvin 21.15.0 [31]. Major microspecies were calculated by means of Marvin at the selected pH of the receptors. Both hesperidin and diosmin have a nearly 1/1 mix of central phenol group ionized/non-ionized at neutral pH. In these cases, docking was performed using each one of the protomers (ionized ligands in the main body of the article, while non-ionized ones may be observed in the Supplementary Material). After microspecies calculation, structures were built up in 3D and geometries were minimized by means of the XED force field [32,33] in Flare. 

#### 2.12.4. Autodock-GPU Docking (AD-GPU)

For AutoDock-GPU, each ligand was transformed in native pdbqt file using a Gasteiger-type charge scheme via MGLtools 1.5.7 [34]. In parallel, the receptor was transformed as well in pdbqt format using a Kollman-type charge scheme with the same software. Receptors were mapped by means of AutoGrid [34], building up a 70 × 70 × 90 points grid of 0.375 Å density, taking the center of the A-F lysozyme cleft as the origin and allowing the box to properly cover the full A-F binding cleft, with a large extension to allow a full scan of any molecule of the aforementioned size in this region. Autodock-GPU [35] was executed in a genetic algorithm modality, with 500 runs, a maximum of 100,000,000 energy evaluations and a maximum of 100,000 of generations, with a starting size population of 300, conforming to an exhaustive search and score exercise. The initial 500 docking results were clustered with a 2Å RMSD and the lowest energy pose was selected.

#### 2.12.5. Flare Lead Finder Docking (LF)

A docking grid was constructed at the center of the A-F HEWL cleft, with a size of 70 × 70 × 90, ensuring again an adequate cover of the cleft and surrounding regions to allow a relaxed conformational scan without grid wall proximity impediments. Lead Finder [36,37] was configured for the normal docking modality, using an “accurate but slow calculation method” (as implemented in Flare), using standard parameters, and both hesperidin and diosmin were docked and scored. The lowest energy pose by means of Lead Finder dG score was retained as the result.

## 3. Results and Discussion

### 3.1. Polyphenols: Content and Distribution

The extracts obtained from orange and lemon peels were characterized by determining their total polyphenolic content and identifying the major components. Concentrations of total phenolics were estimated for lyophilized extracts of peels as well as that of their respective juices, obtained from the corresponding aqueous solutions (30 g/L) collected in triplicate. Quantitative data are expressed in grams of gallic acid equivalents per 100 g of dried extract. Linear regression analysis was performed to obtain equations with the best fit to the points using external calibration with gallic acid. For orange, calibration was obtained from gallic acid aliquots of concentrations 5.92, 11.84, 17.76, 23.68 and 29.6 ppm (Figure S1). Orange peel extract showed a high concentration of phenolics, 4.99 ± 0.37 g gallic acid/100 g dried extract (1.49 g/L). In order to establish the part of the fruit that was a major source of polyphenolic compounds, the total polyphenolic content of the freeze-dried orange juice was also assessed. Calibration was obtained from gallic acid aliquots with the following concentrations: 0.562, 1.184, 1.776, 2.36, and 2.96 ppm (Figure S2). The concentration of total polyphenols found in orange juice was 0.56 ± 0.02 g gallic acid/100 g sample (0.168 g/L). 

For lemon, the total polyphenolic content was also studied for peel and juice, using gallic acid as a standard. For peel extract, calibration was obtained by using the same gallic acid aliquots used for the orange peel extract (Figure S3). The total polyphenolic content quantified for the extract obtained from lemon peel was 5.274 ± 0.103 g gallic acid/100 g extract (1.582 g/L). Likewise, the polyphenolic content of the previously freeze-dried lemon juice was also analyzed. Calibration (Figure S4) was obtained using the same gallic acid aliquots as those used for the orange juice. The concentration of total polyphenols found in lemon juice was 0.481 ± 0.048 g gallic acid/100 g extract (0.144 g/L). 

The fact that the value found for orange juice, 0.56 ± 0.02 g of gallic acid/100 g of sample, was almost nine times lower than that determined for the aqueous extract of the peel, 4.99 ± 0.37 g of gallic acid/100 g of extract, allowed us to choose the latter as the source of polyphenols needed to meet the objectives of this study. In a similar way, the total polyphenolic content of lemon peel presented a value, 5.274 ± 0.103 g of gallic acid/100 g of extract, much higher (>10 times) than that found in the juice of the same fruit (0.481 ± 0.0483 g of gallic acid/100 g of extract). Accordingly, the higher content of phenolic compounds, together with the possibility of using by-products from the agri-food industry, makes fruit peels an ideal source for obtaining these bioactive compounds. 

That said, a broad range of polyphenol content from extracts can be found in bibliographic sources. Such variations can reasonably be attributed to both biotic and abiotic factors (soil composition and pH, solar exposure, humidity, presence and/or persistence of pesticides, etc.). As secondary metabolites, polyphenols may provide protection against plant stress by activating endogenous mechanisms and modulating cellular signaling to different extents [38,39]. Furthermore, the polyphenolic content is markedly influenced by the type of extraction protocol and its operational conditions [40,41,42,43]. Accordingly, and bearing these premises in mind, our results could hardly be compared with others reported in the literature.

### 3.2. Antioxidant Activity of Lemon/Orange Peel Extracts

The well-known ABTS radical cation decolorization assay was applied to dried polyphenol-rich orange and lemon peel extracts [24]. Data were collected in triplicate from aqueous solutions (4.5 g/L) and calibrations were performed using Trolox as an external standard (Figures S5 and S6). Their respective abilities for scavenging free radicals by the phenolic content, 50.92 ± 0.17 mM Trolox/100 g dried extract for orange peel and 49.09 ± 2.57 mM Trolox/100 g dried extract for lemon peel, clearly suggest the remarkable potentiality and similar activity of both citrus extracts as antioxidants. 

It is worth pointing out that antioxidative measurements can depend on the method employed and the experimental conditions. The use of ABTS relative to other radical ions has been a matter of controversy in view of the variable results. A recent analysis, with a focus on ABTS, has revealed that some antioxidants of a phenolic nature can form coupling products with ABTS^•+^, while others can undergo oxidation without coupling. This feature, shared by other antioxidant protocols, may lead to overestimations [44]. In general, however, the ABTS method is reliable and consistent, and adduct-free oxidation pathways are usually substrate-specific, yet influenced by the radical initiator system.

### 3.3. Polyphenolic Patterns of Orange/Lemon Peel Extracts

As mentioned earlier, HPLC analyses of lyophilized extracts show a wide distribution of phenolic compounds. These include structurally simple compounds derived from the flavanone core, together with complex flavone-*O*-glycosides with multiple stereocenters.

**Figure 1:**
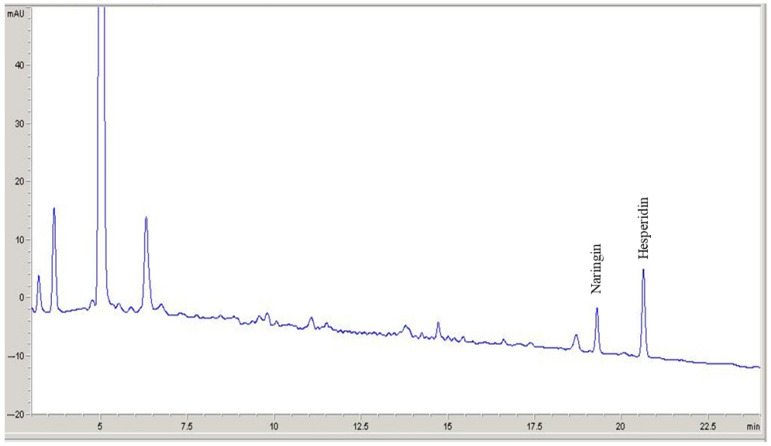
Chromatogram showing the identification of phenolics in orange peel extracts (absorption at 350 nm, diode array detection). Peaks characteristic of naringin and hesperidin were detected at retention times of 19.50 and 20.62 min, respectively.

In the case of orange peel extract, as listed in Table 1, the main constituents are naringin and hesperidin. Figure 1 depicts the chromatographic profile, showing the identification of phenolics in multistandard solutions. These findings align with prior studies on polyphenols found in orange peels, evidencing the presence of hesperidin [45] and naringin [46] as the most abundant species. As noted above, the origin and preservation of phenolic compounds are influenced by biotic factors such as pre-harvest conditions and crop diseases [47], and abiotic ones like the extraction method [41]. There is accumulated evidence that hesperidin, for instance, can undergo oxidative changes, particularly a dehydrogenation reaction resulting in the formation of diosmin [48]. This transformation may justify the increased concentration of this *O*-glycoside detected in our extracts. Curiously (as noted in the tabulated data), anomalous results for naringin, exceeding the parent value, were found, which might be attributed to a related interconversion.

Moreover, literature data show that naringin and hesperidin are the major polyphenols in orange fruits, especially in the edible parts at concentrations of 81.79 ± 0.09 mg/kg and 657.39 ± 0.14 mg/kg, respectively [23]. Likewise, a comprehensive survey on flavanones present in different citrus varieties identified hesperidin, rutin, and naringin as the major components in peels and edible parts [46]. 

**Figure 2:**
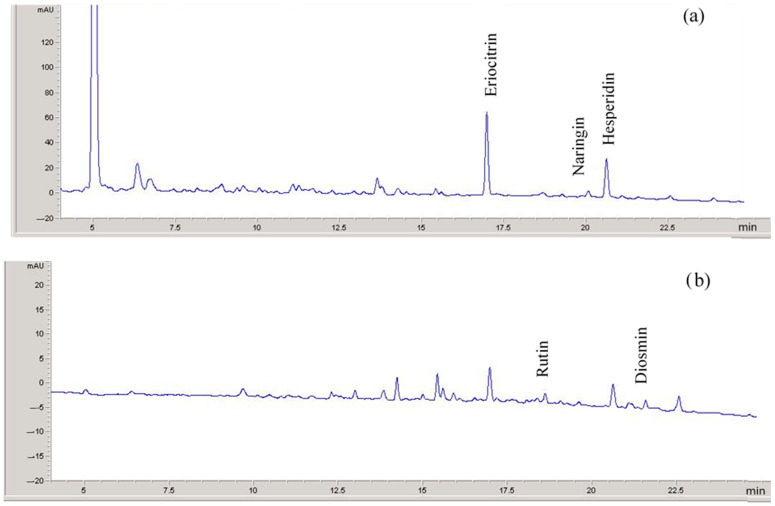
Chromatograms showing the identification of phenolic compounds in lemon peel extracts (absorption at 280 (**a**) and 350 nm (**b**), diode array detection). Peaks characteristic of eriocitrin, naringin, and hesperidin were detected at retention times of 16.90, 19.50, and 20.62 min, respectively (at 280 nm). Peaks characteristic of rutin and diosmin were detected at 18.48 and 21.50 min (at 350 nm).

In the case of lemon peel extract, the major components in decreasing order of concentration are hesperidin, eriocitrin, naringin, rutin and diosmin (Table 2). Figure 2 shows its chromatographic profile with the identification of phenolics in multistandard solutions. Similar to orange extracts, hesperidin is the major polyphenol. This finding is also consistent with previous studies [49], which detected phenolic acids, namely caffeic, ferulic, and chlorogenic acids, as the most abundant species, followed by flavanones such as hesperidin (again the main component among them), hesperetin, and eriocitrin.

### 3.4. Polyphenol–Protein Matrices in the Supernatant Phase–Molecular Interactions from FTIR Spectra

In order to generate polyphenol-fortified protein matrices using extracts from orange and lemon peels and to determine the affinity of such components, the resulting supernatants after formation of the corresponding matrices were screened, using lysozyme and ovalbumin as protein models, which represent the main allergens in cow’s milk. All experiments were carried out at room temperature first. 

Although numerous spectroscopic techniques can be employed to monitor the chemical modifications, which could be both covalent and non-covalent in nature, FTIR spectra, recorded in ATR mode, have proven to be a useful and affordable method, and often provide diagnostic signals for detecting the molecular changes undergone by the protein after interaction with polyphenols and other ligands [25,50]. Thus, variations in peak absorptions and band intensity typically reflect changes in secondary protein structure and local conformation, where hydrogen bonding can be significantly altered. The most salient absorptions for spectral monitoring are the amide I (1630–1700 cm^−1^), amide II (1510–1570 cm^−1^), and amide III (1230–1330 cm^−1^) bands. The amide I and II bands are usually intense and reflect vibrations of the peptide backbone. The former results from C=O stretching, whereas the amide II band combines both N-H bending and C-N stretching vibrations. These bending and stretching modes also contribute to the amide III band, although its intensity is much lower. Unambiguous identification of the latter often requires converting the labile N-H bond into an N-D bond through deuterium exchange. Accordingly, our investigation focused on the amide I and II bands, whose variations correlate well with conformational changes in the parent protein structures. Despite all proteins being lyophilized prior to matrix formation, the presence of residual moisture could not be overlooked. Even though the OH group exhibits a broad and intense stretching band above 3100 cm^−1^, water (H-O-H) shows bending vibration at approximately 1645 cm^−1^, coincidental with the amide I band [25].

Table 3 shows the lysozyme:polyphenol interaction percentages, as quantified by HPLC after analysis of the supernatants resulting from the corresponding matrices (protein:extract ratios 1:1, 1:2, 2:1) with the two major polyphenols identified in the orange peel extract. For all of the ratios checked, hesperidin, in line with that observed for the ovalbumin matrices with the same extract (Table S1), was found to be the polyphenol that interacted most strongly with lysozyme. For both proteins, ovalbumin and lysozyme, the 2:1 ratio showed the highest percentages of interaction involving the major polyphenols in the orange extract.

**Figure 3:**
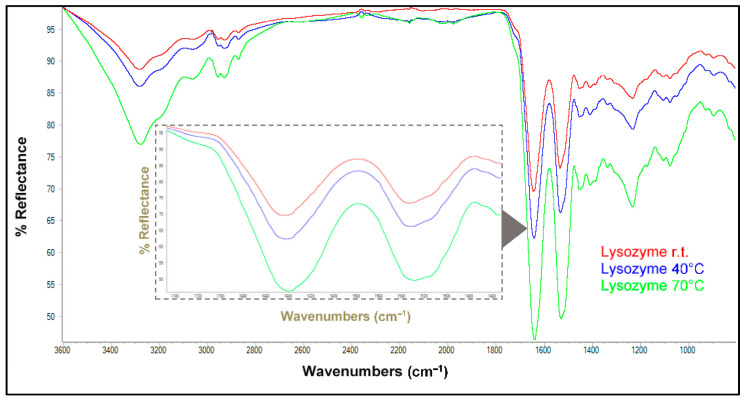
ATR-FTIR spectra recorded for lysozyme at room temperature, 40 °C, and 70 °C. The inner inset shows an enlargement of the absorptions corresponding to amide bands I and II (1200–1800 cm^−1^).

Assuming the possibility of H-bonds between the protein skeleton and water molecules, which may induce conformational changes, ATR-IR spectra of lyophilized ovalbumin and lysozyme, previously dissolved in phosphate-buffered saline (PBS), were recorded. As expected, an increase in temperature led to changes in the bands of interest for both lysozyme (Figure 3) and ovalbumin (Figure S7). In order to disentangle the effect of the interactions with extracts and/or individual polyphenols on the structure of both proteins, the experiments described below were carried out at room temperature. 

**Figure 4:**
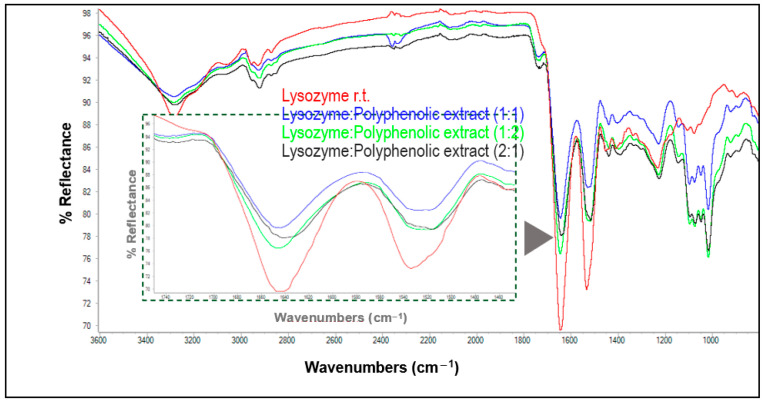
ATR-FTIR spectra of unmodified lysozyme and its matrices with polyphenols from orange peels at room temperature. The inner inset shows an enlargement of the absorptions corresponding to amide bands I and II (1200–1800 cm^−1^).

FTIR spectra for matrices obtained from dissolved lysozyme at room temperature and the orange peel extract at different ratios, as well as the spectrum of the uncombined protein, are depicted in Figure 4. The intensities of the bands in the region of amide bonds I and II show a clear decrease relative to those of the uncombined protein. It appears that the spectrum corresponding to the 1:1 lysozyme:extract matrix showed the most pronounced change with respect to the native lysozyme, followed by the spectrum of the 2:1 matrix (which showed the highest degree of interaction according to HPLC; see Table 3), and, finally, the 1:2 matrix, which exhibited a smaller deviation, although it still showed a remarkable change. For ovalbumin, the protein:extract ratio seemed to have little or no influence on the changes in band intensities (Figure S8, Table S1).

**Figure 5:**
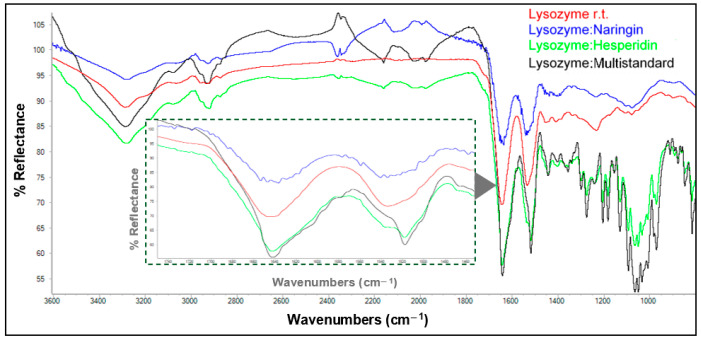
ATR-FTIR spectra of lysozyme and its matrices with pure polyphenols and a multistandard solution. The inner inset shows an enlargement of the absorptions corresponding to amide bands I and II (1200–1800 cm^−1^).

These experiments were performed at different ratios and temperatures, i.e., room temperature, 40 °C and 70 °C. Evaluation of the interaction percentages indicate that the 2:1 ratio shows the greatest degree of interaction between these proteins and the polyphenols, as noted earlier, and hence, this parameter and room temperature were chosen as working conditions to evaluate the individual and multistandard polyphenol interactions with both proteins. Subsequently and under such conditions, new matrices were generated between lysozyme and the main polyphenols present in the orange peel extract, naringin and hesperidin, pure, combined as a multistandard and doubling the concentration, respecting in all cases the concentrations at which these polyphenols were found in the citrus peel extract. Figure 5 shows the IR-ATR spectra of the matrices formed by lysozyme in combination with naringin and hesperidin, both individually and as part of the multistandard solution.

The bands of the lysozyme–naringin matrix spectra showed a lower intensity than that observed for the bands of the uncombined protein spectrum. On the other hand, the bands corresponding to the lysozyme–hesperidin matrix, as well as the multistandard solution, showed higher intensities than those of native lysozyme. In addition, the bands of the latter were more intense than the bands of the hesperidin matrix. Table 4 depicts the percentages of interaction involving lysozyme with both polyphenols. In all cases, hesperidin was found to be the polyphenol that interacted to the highest extent.

**Figure 6:**
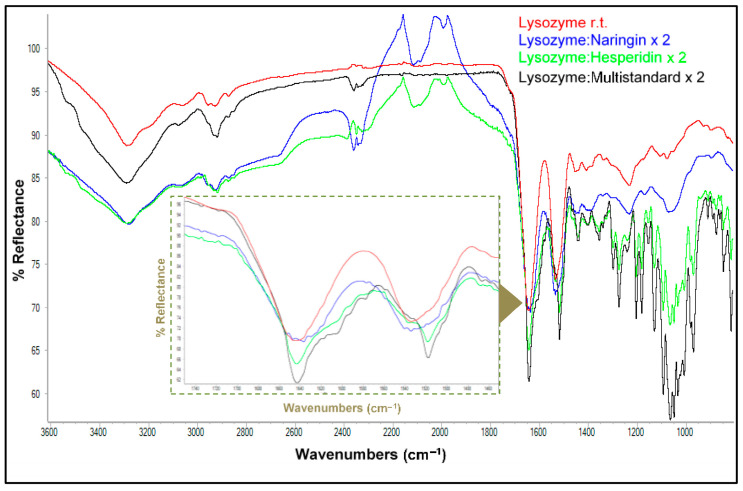
ATR-FTIR spectra of lysozyme and its matrices with pure polyphenols and a multistandard solution with double concentrations of phenolics. The inner inset shows an enlargement of the absorptions corresponding to amide bands I and II (1200–1800 cm^−1^).

For ovalbumin (Figure S9), the native protein showed the spectrum having the lowest intensity absorptions. The bands relative to the matrices obtained independently with naringin and hesperidin showed a similar degree of modification. However, the matrix formed by ovalbumin and the multistandard mixture showed a higher intensity than the rest. This fact could point to a possible synergistic effect between both polyphenols. In all of the matrices tested, hesperidin was found to be the polyphenol that interacts with the protein to the greater extent (Table S3). Finally, spectra were recorded for matrices generated from lysozyme and the major polyphenols detected in the orange peel extract, both individually and combined as a multistandard mixture, in all cases at twice the concentration as noted above (Figure 6).

Spectral bands corresponding to the protein alone and the matrices formed with lysozyme and naringin or hesperidin showed similar intensities. Uncombined lysozyme had the least intense bands, followed by the bands of lysozyme–naringin, lysozyme–hesperidin, and lysozyme–multistandard matrices, the latter showing noticeable, yet minor, differences between 1200 and 1800 cm^−1^. Also, a slight narrowing was observed in the bands corresponding to the polyphenolic matrices with respect to the lysozyme bands. Table 5 shows the interaction percentages for all matrices. Once again, hesperidin was found to be the polyphenol that interacted with lysozyme to the largest extent.

Unlike the previous case (Figure S9), the study of ovalbumin matrices formed with phenolic compounds at the double concentration (Figure S10) found that the spectrum showing lower-intensity bands was the one corresponding to the matrix formed with hesperidin. In contrast, the matrix formed with naringin did show an increase in intensity, even more significant in the case of the multistandard solution at twice the concentration. This could indicate that the protein:polyphenol interaction is not necessarily concentration-dependent, although a synergistic effect is apparent. Table S4 shows the percentages of interaction, with hesperidin the polyphenol that interacts with ovalbumin to a greater extent. 

On the other hand, the interaction percentages involving lysozyme (Table 6) and ovalbumin (Table S2) with the main polyphenols identified in the lemon peel extract indicate that, for all of the ratios tested, diosmin showed the strongest interaction (*ca*. 100%), together with rutin in the case of ovalbumin (2:1 ratio). As described for the polyphenols present in the orange peel extract, again the 2:1 ratio provided the highest level of interaction with the two egg proteins.

**Figure 7:**
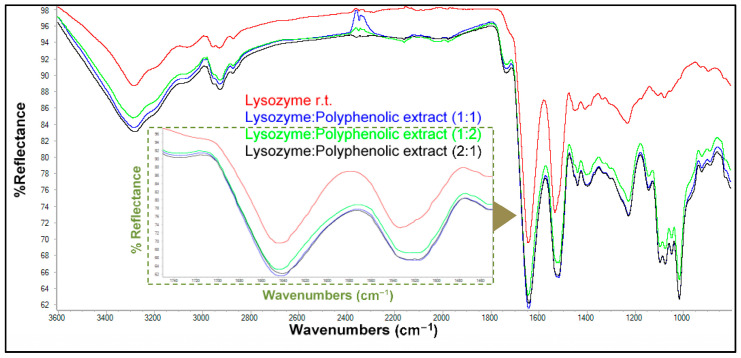
ATR-FTIR spectra of native lysozyme and its matrices with polyphenols from lemon peels at room temperature. The inner inset shows an enlargement of the absorptions corresponding to amide bands I and II (1200–1800 cm^−1^).

Therefore, using the same working conditions as above (room temperature at 2:1 ratio), new matrices were generated between lysozyme or ovalbumin with the polyphenols present in lemon peel extract. The spectra of native lysozyme, as well as those of the hybrid matrices, are shown in Figure 7.

As seen across the region between 1200 and 1800 cm^−1^, the lysozyme spectrum showed low-intensity bands with respect to the spectra obtained with the rest of peel extracts, which, however, did not exhibit any significant differences. This trend seems to be contrary to that observed in Figure 4, where the absorption bands relative to the protein spectrum had a higher intensity than those of the matrices formed with the orange peel extract. As previously mentioned, diosmin showed the highest interaction percentage (Table 6). 

For ovalbumin (Figure S11), there is a decrease in band intensity for all matrices when comparing to that of the native protein. In this case, the bands due to the 1:1 matrix are close to the bands of the native protein spectrum, with the 1:2 ratio exhibiting the greatest difference and placing the 2:1 ratio in the middle ground. This behavior is similar to that observed for the lysozyme matrices, with diosmin being again the polyphenol with the highest percentage of interaction (Table S2).

**Figure 8:**
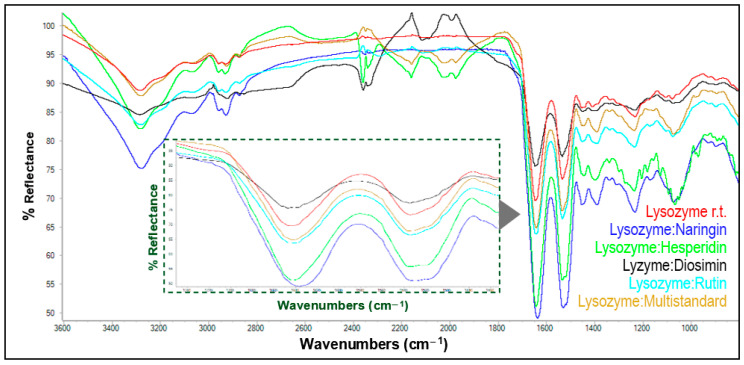
ATR-FTIR spectra of lysozyme and its matrices with pure polyphenols and a multistandard solution. The inner inset shows an enlargement of the absorptions corresponding to amide bands I and II (1200–1800 cm^−1^).

Consistent with the present methodology, new matrices involving lysozyme at room temperature and the main polyphenols from the lemon peel extract (2:1 ratio) were then developed, alone, combined as a multistandard solution, and doubling the concentration, using the identical concentration at which these polyphenols were found in the extract. The corresponding eriocitrin matrices were not generated owing to the exorbitant price of this polyphenol. Likewise, FTIR-ATR spectra were recorded for all of the lysozyme matrices as shown in Figure 8.

For such matrices and within the region corresponding to amide bands I and II, most absorptions show an increased intensity with respect to those of the uncombined protein; the naringin matrix actually exhibits the most intense bands, followed by the hesperidin, rutin, and multistandard matrices. On the other hand, it is striking that peaks corresponding to the matrix with diosmin show a slight decrease in intensity with respect to the bands of the lysozyme spectrum. In this case, contrary to what was observed for the matrices obtained from orange polyphenols (Figure 5 and Figure 6), no synergistic effect seems to be observed. Table 7 shows the interaction percentages involving lysozyme and the main pure polyphenols identified in the lemon peel extract, individually and forming a multistandard solution, as usual. The strongest interaction is shown by diosmin, which reached 100% when combined alone with the protein. In the multistandard matrix, the highest interaction degree was observed for hesperidin, followed by diosmin and rutin.

**Figure 9:**
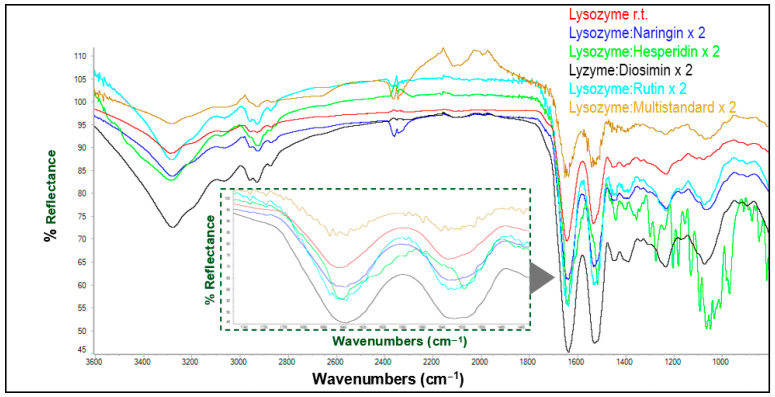
ATR-FTIR spectra of lysozyme and its matrices with pure polyphenols and a multistandard solution with double concentrations of phenolics. The inner inset shows an enlargement of the absorptions corresponding to amide bonds I and II (1200–1800 cm^−1^).

In the case of ovalbumin, as depicted in Figure S12, the native protein showed the lower-intensity bands. In contrast, the most intense bands correspond to the spectra of the hesperidin and diosmin matrices. The bands corresponding to the multistandard mixture had an intermediate intensity. Again, this suggests the absence of a synergistic effect. According to the trend observed in the lysozyme matrices, diosmin, followed by rutin, was the polyphenol that interacted to the greatest extent, although this order was swapped in the case of the multistandard solution (Table S5). Finally, spectra for the matrices formed between lysozyme and the polyphenols present in the lemon peel extracts, both individually and combined (multistandard) at twice the concentration, are shown in Figure 9.

It is noteworthy that the spectrum for the diosmin matrix shows the most intense bands, followed by those corresponding to hesperidin, rutin and naringin, all very close in terms of intensity. The bands due to the uncombined protein show a lower intensity than the aforementioned matrices, although they are higher than those shown by the matrix formed with the multistandard solution, which is the least intense by far. Similar to what was shown in Table 7, diosmin, followed by rutin, was again the polyphenol that individually interacted strongly. For the multistandard matrix, this role was played by hesperidin (Table 8).

In the corresponding ovalbumin spectra (Figure S13), following a similar trend as that shown in Figure S12, the bands assigned to the diosmin matrix exhibit the most intense bands. In this case, the bands due to the hesperidin matrix do not differ appreciably and are grouped into the absorption bands shown by other matrices. In agreement with the HPLC measurements, diosmin was newly, followed by rutin, the polyphenol with the highest interaction percentage. Lastly, the overall interaction seemed to be somewhat stronger when the polyphenols were forming the multistandard solution, which could indicate a possible synergistic effect (Table S6).

### 3.5. Molecular Docking Campaign of Lysozyme–Polyphenol Binding

In light of the relevant experimental outcomes for hesperidin and diosmin, in silico investigations to describe the lysozyme–polyphenol binding nature were envisaged. To properly address the virtual calculations, both an analysis of lysozyme structural diversity and the docking background of lysozyme–polyphenol interactions were explored.

#### 3.5.1. Landscape of 3D Reported Lysozyme Structures

**Figure 10:**
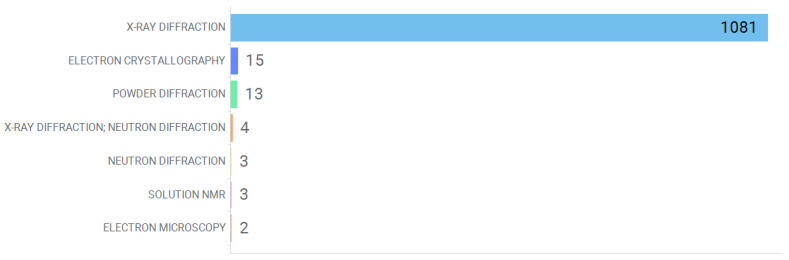
Distribution of PDB experimental method for HEWL deposited structures.

Hen’s egg white lysozyme (HEWL, EC.3.2.1.17) has been extensively studied and largely crystallized. An RCSB-PDB [28] advanced query via Vernalis Research Knime nodes [27] retrieved a total of 1213 3D structures when searching by Uniprot code P00698 (search date 9 July 2024) [51]. Among these, 1121 structures were available as standard .pdb files, which were downloaded and analyzed initially by means of the PDB downloader and PDB property extractor aforementioned at Vernalis Research nodes. Figure 10 summarizes the PDB structures distributed by the experimental characterization method, where standard X-ray diffraction dominates the structural characterization landscape, with minority alternative characterization techniques like solution NMR, neutron diffraction or electron microscopy. 

**Figure 11:**
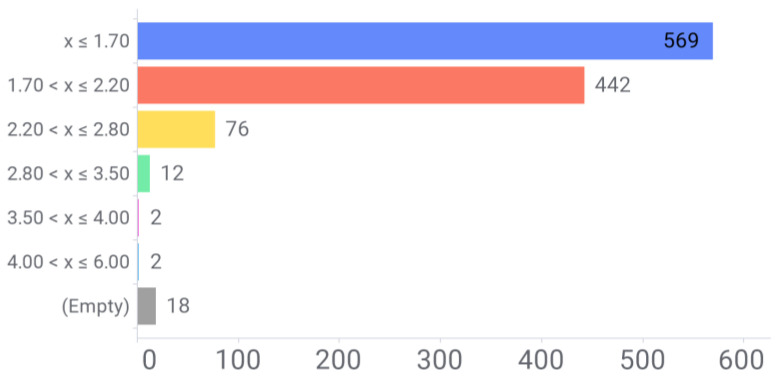
Breakdown of PDB structures by experimental resolution. Empty data refers to those records without annotated resolution in the .pdb files.

Our next stage was extracting basic crystallization data like resolution, pH and the presence of co-crystallized ligands. A simple analysis of the HEWL structure dataset resolution is depicted in Figure 11. The vast majority of records possess highly resolved structures, with optimal resolutions below 2.20 Å, allowing most atoms to be observed very clearly.

**Figure 12:**
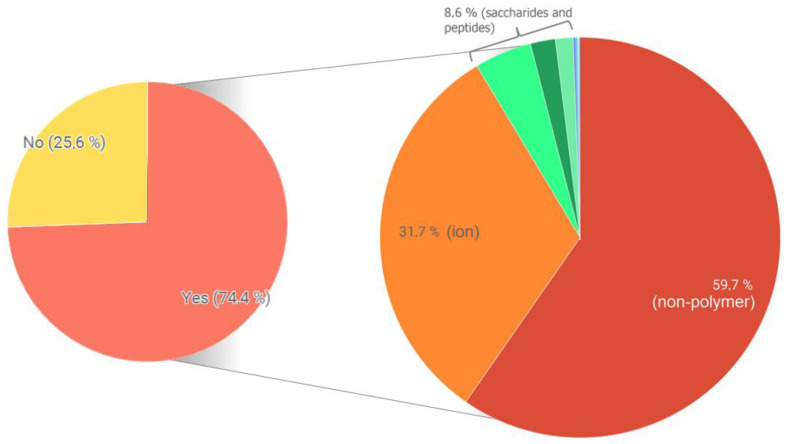
Left-hand side: Breakdown of PDB structures by presence of co-crystallized ligands. Right-hand side: PDB-like classification of ligands and cumulative percentage.

Figure 12 shows two informative pie charts around the ligand space of PDB HWEL. Almost 25% of the crystallized proteins are apo structures, while the remaining 75% contain some ligands. PDB files normally label as ligands different chemical entities: buffer molecules, solution and metalloprotein ions, drug-like compounds, etc. Further decomposition of this space by PDB ligand classification revealed that 31.7% of the cumulative ligands are in fact metal, oxoacid and organic ions. Nearly 60% of the cumulative ligands are a non-polymer type: buffers, druglike molecules and coordination complexes. Inside this group, flavonoid-based structures are not present. The remaining 8.6% of the ligand dataset are a collection of peptides and saccharides.

**Figure 13:**
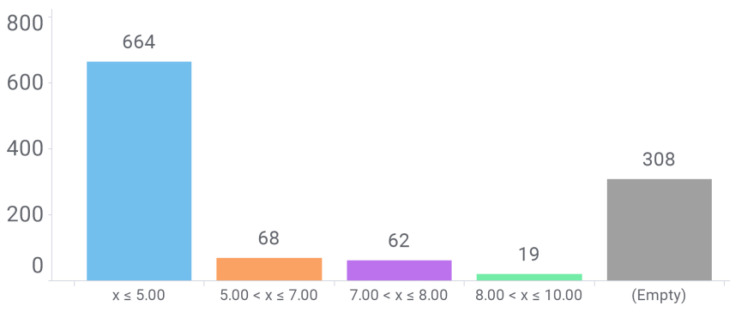
Distribution of PDB structures by experimental pH of crystallization. Empty data refers to those records without annotated pH in the .pdb files.

The last piece of information involves the experimental pH of protein crystallization (Figure 13). There is a large group of structures (308 of 1121) without any pH information contained in the .pdb files. This reflects some information, including pH among them, which is not properly covered in these files, requiring deep analysis of the materials and methods of the related articles. Due to the scale of pH-based uncovered structures, this was not performed. Most of the datasets were reported to be crystallized at acidic conditions (pH below 5) with small sets being grown under moderately acidic (pH between 5–7), neutral (pH between 7–8) and moderately basic (pH between 8–10) conditions.

Based on these analyses, some preliminary conclusions may be obtained: (a) hen’s egg white lysozyme (HEWL) presents 1121 PDB records, most of them generated by x-ray diffraction techniques, with a high degree of structural resolution (below 2.2 Å). (b) Three out of four structures contain at least one crystallized ligand. Cumulative decomposition analysis revealed nearly 32% of these are ions, and almost 60% are small molecule entities such as druglike molecules, coordination compounds or buffers. Diverse peptides and saccharides complete the ligands space. No flavonoid–lysozyme complexes are present. (c) Circa 300 .pdb files were recorded without experimental pH conditions. Over 600 entries were largely crystallized in moderately acidic pH media (below 5), together with three pH groups (weakly acidic, neutral and weakly basic) with discrete numbers. 

#### 3.5.2. Molecular Docking Precedents of Lysozyme

Lysozyme-based molecular docking studies are numerous in the literature, covering purposes like amyloidosis [52,53,54,55,56,57,58,59,60,61,62,63,64,65,66,67,68,69,70,71,72,73,74,75,76,77,78,79], diffusion studies [80], isoforms comparison [81], protein photodegradation [82,83], or just small molecule binding topology descriptions. The latter is justified in terms of lysozyme’s well-known capability as a drug delivery carrier, due to its natural abundance, small size or higher thermostability. The scientific literature contains a plethora of descriptive works reporting the ligand binding affinities of drug molecules [84,85,86,87], food additives, contaminants and natural products to HEWL. Among the natural products tested in docking exercises, flavonoid families are recurrent in exploring the conformational features of binding to lysozyme. Table 9 summarizes literature precedents together with zinc database identifiers and lysozyme pdb structures employed in molecular docking.

Hesperidin and diosmin were among the flavonoids characterized through conformational exploration via docking. Furkan et al. [64] described diosmin–HEWL effects from an experimental point of view (ANS and intrinsic fluorescence, Rayleigh scattering, circular dichroism and transmission electron microscopy, among others) and computationally via tandem molecular docking–molecular dynamics simulations. For the computer assisted part, the authors used the 2LYZ structure as a rigid body and diosmin as a flexible entity, running a standard Autodock docking run, retaining the lowest energy conformation as the selected topology to be promoted in the molecular dynamics. Ratnaparkhi et al. [76] investigated hesperidin–HEWL complexes by means of a similar collection of empiric techniques (synchronous and intrinsic fluorescence, circular dichroism and fibrillar aggregation) and parallel molecular docking–molecular dynamics calculations. The researchers adopted a single point mutant 2WAR receptor (E35Q) and performed a Glide XP docking run where the lowest energy pose was sampled in a 10 ns MD simulation.

Despite these antecedents, some questions remained unanswered in light of the results of both studies. Specifically:

- Conformational landscape: each of the aforementioned studies contemplated just one receptor conformation in rigid modality and the docking result captured the lowest energy topology. No further discussion about the HEWL conformational landscape or alternative ligand binding modes have been produced. As any molecular dynamics execution depends on the starting coordinates, the simulation course is heavily dependent on such initial states. Diosmin post-docking MD simulations are scarcely described, referring to the overall progress of simulation, number of hydrogen bonds, and receptor fluctuations, without finding a description of the conformational change of the binding mode upon the progression of the simulation. In this context, it is difficult to determine from the study by Furkan et al. essential data like the conservation of the starting binding pose (i.e., the docking selected pose) or the binding pocket conformational evolution. Regarding the hesperidin post-docking MD simulations, Ratnaparkhi et al. outlined the critical interacting residues, some being the original results from the docking output. However, the overall evolution along the simulation remains unclear as well as the identity of the receptor residues interacting by H-bonding with the ligand.
- Receptor mutants: Ratnaparkhi et al.’s in silico calculations operated on the 2WAR single point mutant HEWL (E35Q) [96], where the critical glutamate residue of the enzymatic center was swapped for glutamine. This point change is in the binding pocket, at the center of the docking box described. The authors did not discuss at any point the mutant nature of the receptor or the purpose of that selection. It is highly possible that such a profound change (polar charged lateral chain to simply polar) may impact a small molecule non-covalent attachment compared with native HEWL. It is arguably better to use a non-native structure if the ultimate goal is evaluating the anti-aggregation capacity of hesperidin and subsequent comparisons with experiments employing natural HEWL.
- Absence of experimentally found epitopes: the experimental assays covered describe the overall binding conclusions (i.e., the influence of Tyr or Trp residues on the fluorescence) but no specific epitopes, ligand ring flips or sugar unit alignments that may guide the design and selection of appropriate in silico studies in future.
- Receptor pH: as the endpoint is monitoring the hesperidin/diosmin–lysozyme binding impact ex vivo, a study is required that covers the acidic, basic and neutral forms of the protein.

Conclusively, the reported in silico hesperidin/diosmin–lysozyme models do not suffice to address the preceding questions or, ultimately, the conformational landscape of the receptor and such flexible molecules.

#### 3.5.3. Consensus Molecular Docking Exploration of Hesperidin and Diosmin

Based on the above observations, a deeper in silico docking exercise is required to address the true binding nature of hesperidin/diosmin with HEWL. To confidently explore the conformational space of these systems, a consensus docking is envisaged using a 14-set of lysozyme crystallized structures at different pHs from the PDB repository and two distinct docking engines, Autodock-GPU and Flare. Using a larger number of receptors coupled with different docking engines is a solid strategy to address the conformational conundrum of these systems.

Table 10 summarizes the PDB 3D structures used as rigid body receptors in the consensus docking exercise, being appropriately prepared in terms of protonation states according to the experimental crystallized pH. The selection criteria of these structures were based on terms of pH (acidic, neutral and basic), high resolution, co-crystallized ligand or extensive usage in the literature (6LYZ, 2LYZ, 1HEW, 1JPO and 1DPX).

**Figure 14:**
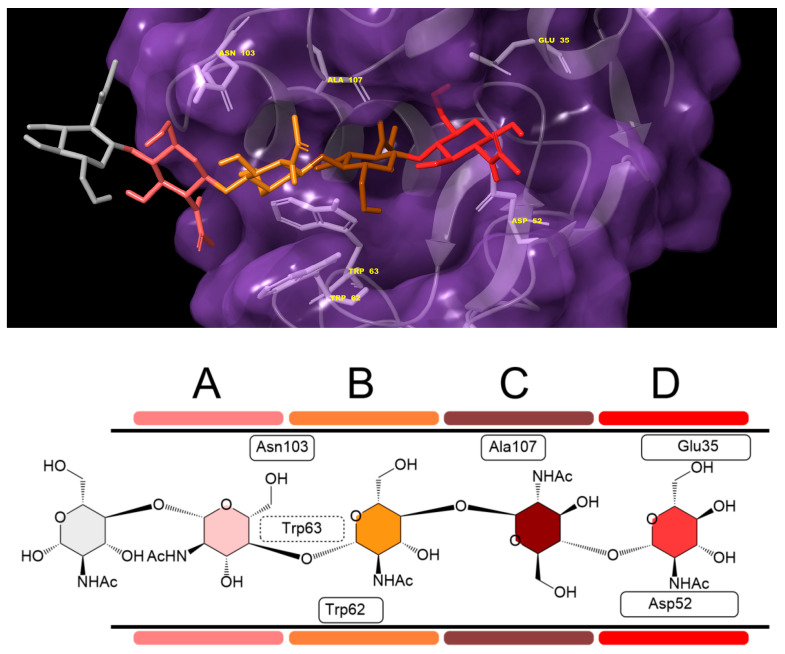
Depiction of the catalytic center of HEWL allocating polysaccharide in subregions A–D. Top image shows 3D arrangement with key residues. Bottom image depicts a 2D diagram that will serve as a docking results template.

Receptor and hesperidine/diosmin structures were prepared accordingly and subjected to molecular docking executions following the methodologies described in Section 2.12. As noted, the docking region was restricted to the catalytic cleft, where six saccharide units are accommodated in the subregions A–F. The docking results in particular show the ligand arrangements in subregions A–D (Figure 14). 

**Figure 15:**
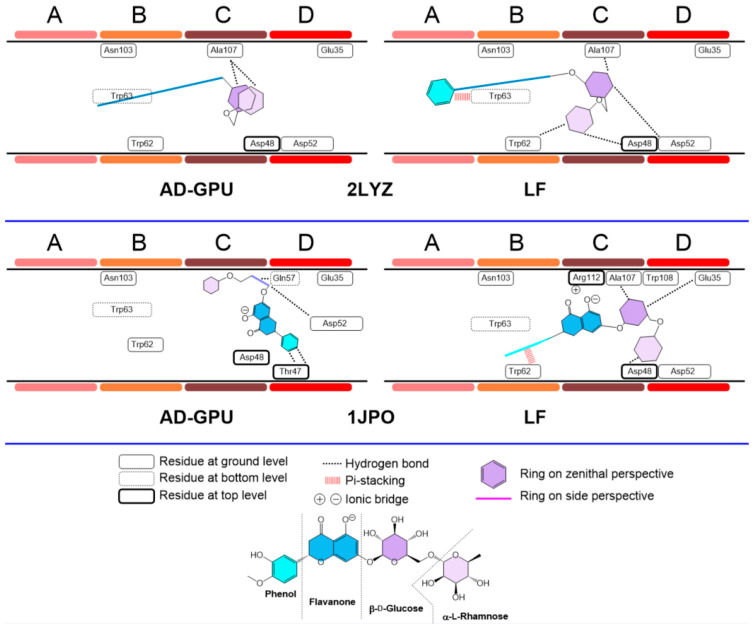
Hesperidin–lysozyme binding topologies (neutral pH, ionized) with 2LYZ and 1JPO, showing the relative position of the ligand to the ABCD pocket clefts.

**Figure 16:**
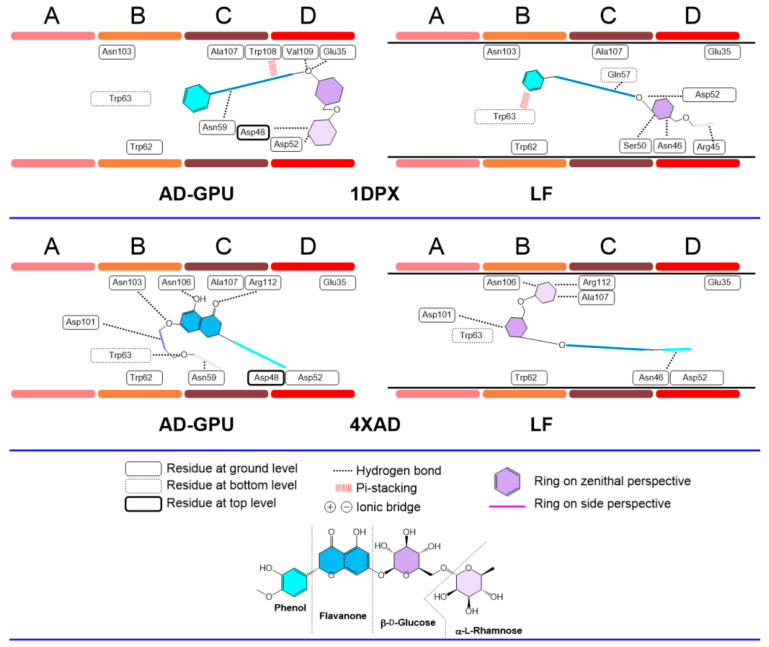
Hesperidin–lysozyme binding topologies (acidic pH) with 1DPX and 4XAD, showing the relative position of the ligand to the ABCD pocket clefts.

**Figure 17:**
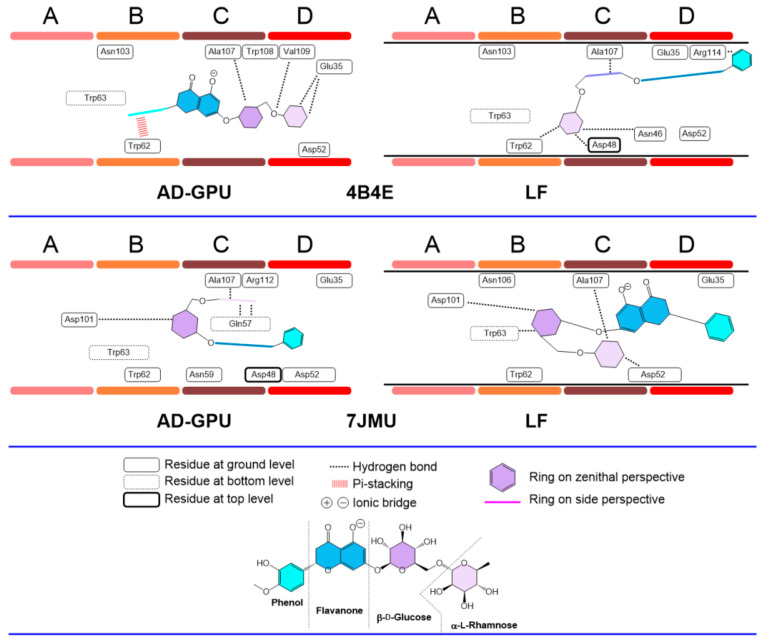
Hesperidin–lysozyme binding topologies (basic pH) with 4B4E and 7JMU, showing the relative position of the ligand to the ABCD pocket clefts.

The hesperidin consensus docking results for receptors 2LYZ and 1JPO (neutral pH, ionized); 1DPX and 4XAD (acidic pH); and 4B4E and 7JMU (basic pH) are represented in Figure 15, Figure 16 and Figure 17. These are representative of the whole 14-pack (for the remaining results, see the supporting information). In general, hesperidin exhibits different configurations (i.e., position, orientation and conformation) depending on the selected receptor, protomer and docking engine. Flavanone/phenol moieties are not restricted to the A-B domains, as previously reported [76], with stacking-like interactions with Trp62−63, with cases like 4XAD and 7JMU showing alternative arrangements, with the sugar rings placed in the B-C regions while the aromatic ones are allocated to the C-D domains. The polyhydroxylated nature of hesperidin makes hydrogen bonding its primary interaction modality. Hydrogen bond schemes differ between results: in cases like 1JPO (Flare), 2LYZ (Flare), 1DPX (both AD-GPU and Flare) and 4B4E (AD-GPU), the key pattern residues are those from C-D domains, frequently including Glu35, Asp52, and Ala107. Internal residues like Gln57 are also subjected to hydrogen bonding, like 1DPX (Flare) and 7JMU (AD-GPU). Sugar ring conformations are dominated by boat-like conformations, with both six-membered rings and flavanone scaffolds found frequently deep on the cleft to maximize the hydrogen bonding interactions.

**Figure 18:**
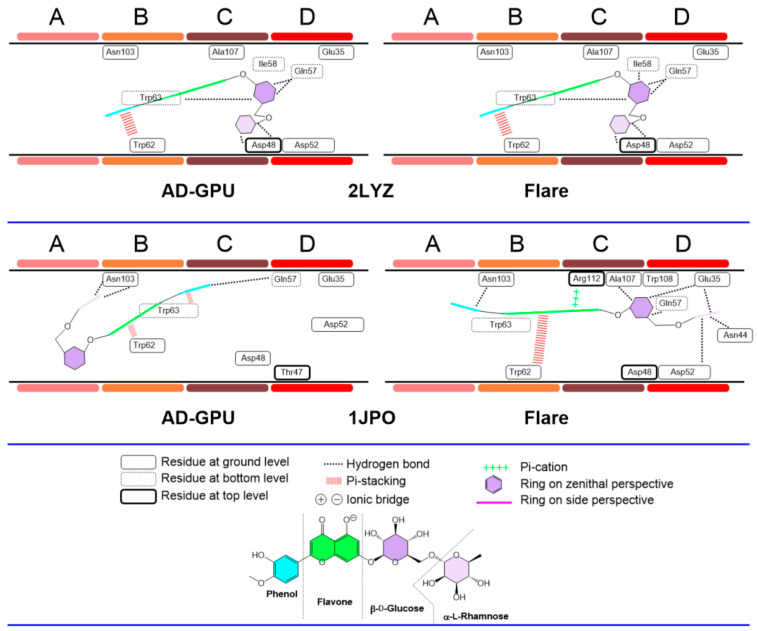
Diosmin–lysozyme binding topologies (neutral pH, ionized) with 2LYZ and 1JPO, showing the relative position of the ligand to the ABCD pocket clefts.

**Figure 19:**
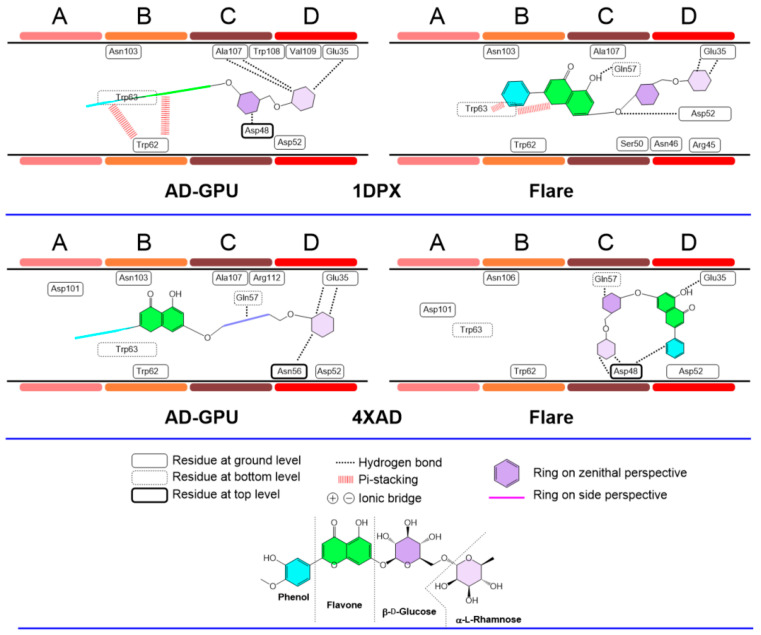
Diosmin–lysozyme binding topologies (acidic pH) with 1DPX and 4XAD, showing the relative position of the ligand to the ABCD pocket clefts.

**Figure 20:**
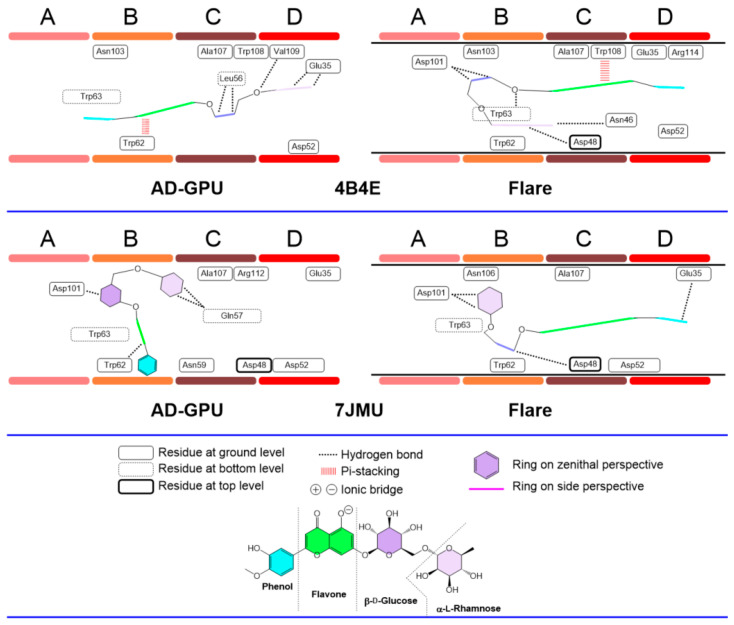
Diosmin–lysozyme binding topologies (basic pH) with 4B4E and 7JMU, showing the relative position of the ligand to the ABCD pocket clefts.

The diosmin consensus docking outcomes for receptors 2LYZ and 1JPO (neutral pH, ionized); 1DPX and 4XAD (acidic pH); and 4B4E and 7JMU (basic pH) are shown in Figure 18, Figure 19 and Figure 20. These are representative of the whole 14-pack (for the remaining results, see supporting information, Figures S14–S21). Similarly to hesperidin, diosmin deploys dissimilar binding topologies to HEWL. Scaffold desaturation (flavanone to flavone) has the impact of augmenting the planarity of the non-sugar region. This structural effect results in an increased number of cases where the aromatic rings are coplanar, as well as the prevalence of ligand accommodation on its edge in the A-D clefts, i.e., aromatic rings are observed with edge-like arrangements more than zenithal ones. This feature makes it more likely to find pi-stacking and pi-cation type interactions with Trp62−63 and Trp108, like cases 2LYZ, 1JPO, 1DPX and 4B4E. Sugar ring conformations present, on the other hand, prevalent chair-like conformations. A hypothesis about this ring swap is the frequent edge-like allocations of diosmin, restricting aromatic rings to interact via pi-stacking to Trp62−63 quite frequently, hence orienting sugars to shallow spots. This collection of features has not been observed before [64].

To sum up, molecular docking studies on hesperidin/diosmin binding to HEWL indicate that both ligands present different binding topologies depending on the receptor, pH and docking engines employed. Slight receptor changes have a profound impact on binding modes due to the flexibility and polyhydroxylated nature of the natural products involved. Additionally, flavanone to flavone scaffold hopping (hesperidin to diosmin) augments the general planarity of the system, finding more edge-like arrangements of the aromatic rings and influencing a shallow allocation of sugars, which turns boat-like sugar conformations into chair-type ones.

Given these facts, molecular docking studies are capable of envisaging a complex, multifaceted topic, contrary to the overall literature precedents, detailing a single-receptor, single-pose avenue, despite recovering the flexibility with subsequent molecular dynamics simulations. Overcoming this challenge requires an expansion of these docking studies in two avenues: (a) progressing the overall topologies to all-atom molecular dynamics simulations, where topological evolution is monitored to observe convergence between starting poses as well as to perform accurate energy evaluations (like MM-GBSA or FMO), and (b) expansion of the 14-lot receptor to a larger collective in order to observe convergence at the molecular docking level.

### 3.6. Determination of IgE Reactivity of the Protein-Polyphenol Matrix

**Figure 21:**
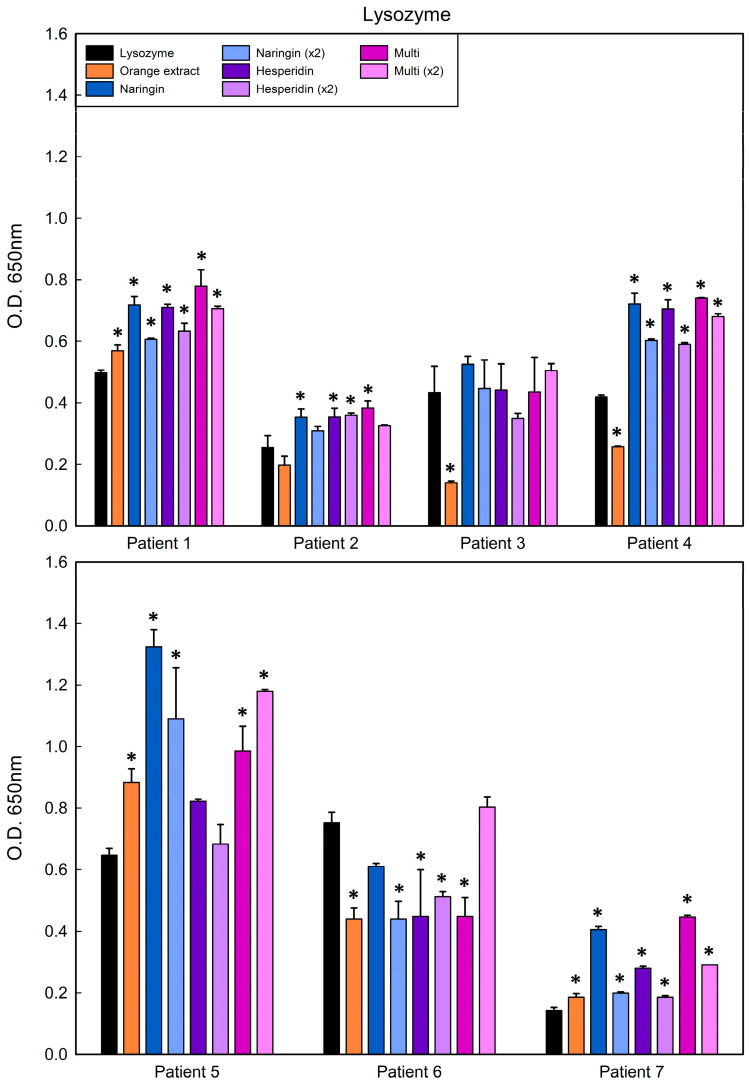
Indirect ELISA using sera from seven patients allergic to lysozyme, using the native protein (black) and different protein–orange polyphenol matrices. Statistically significant differences between the matrix and native protein are indicated with an asterisk (*p* < 0.05).

**Figure 22:**
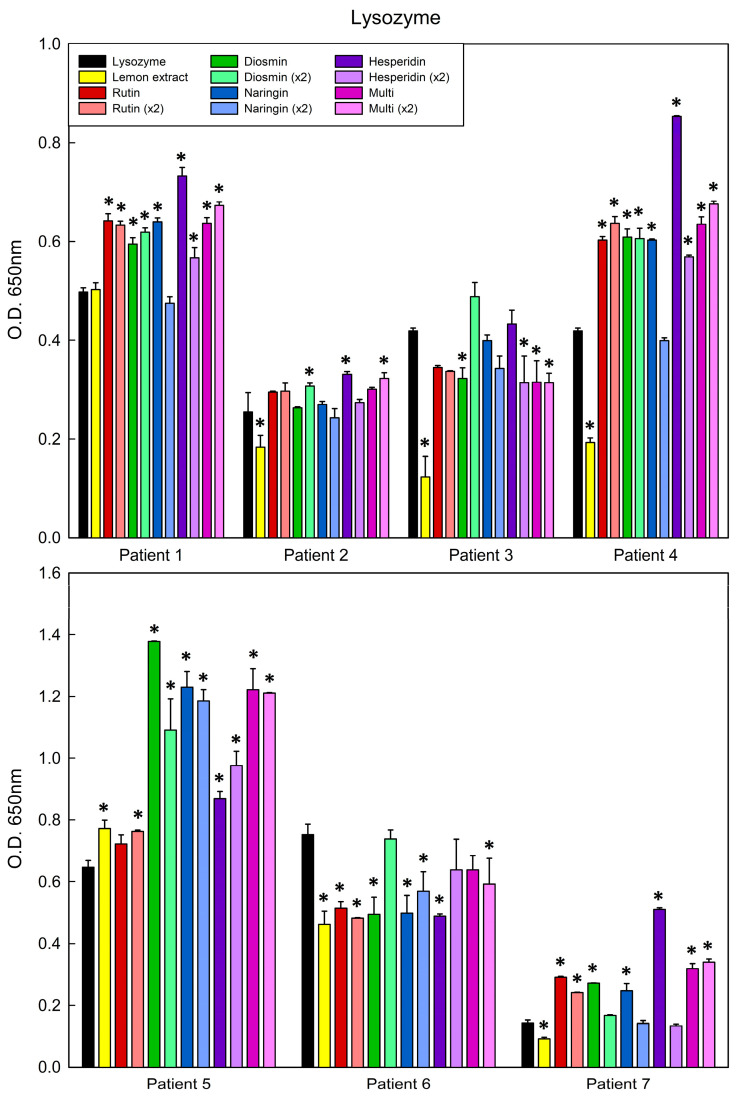
Indirect ELISA using sera from seven patients allergic to lysozyme, using the native protein (black) and different protein–lemon polyphenol matrices. Statistically significant differences between the matrix and native protein are indicated with an asterisk (*p* < 0.05).

To determine if polyphenol-bound protein matrices modify the recognition of the allergen, sera from eight allergic patients (ranging ages 10–77) were evaluated. All of the patients experienced severe, and in some cases life-threatening, symptoms after ingesting hen’s eggs (Table 11). Therefore, it is desirable that the matrix shows less (or the same at least) ability to bind specific IgE from allergic patient sera, thereby reducing the risk of an adverse reaction when the patient is exposed to the allergen–polyphenol matrix. All patients showed serum specific IgE (s-IgE) to ovalbumin (Gal d 2), and seven to both ovalbumin and lysozyme (Gal d 4). The results from such clinical assessments with lysozyme and ovalbumin are displayed in Figure 21 and Figure 22 and Figures S22 and S23, respectively.

Matrices formed by native lysozyme and orange peel extract showed a decrease (but not an impairment) of IgE binding in four sera out of seven allergic patients, being statistically significant in three of them. On the other hand, matrices consisting of native lysozyme and naringin, hesperidin or multistandard solution decreased IgE only in patient No. 6 (Figure 10). 

In the case of matrices formed between the same extract and ovalbumin, immunoassay results were promising with respect to hesperidin (four out of eight patients) with a statistically significant decrease in IgE binding compared to native protein controls. However, patients No. 3, 5, and 6 showed higher IgE binding to ovalbumin–polyphenol matrices than the other patients (Figure S22).

Matrices formed by merging lysozyme with lemon peel polyphenols (extract and pure components) exhibited slightly better results than those of orange peel; actually, five out of seven patients had decreased IgE binding when lysozyme was combined with lemon extract (Figure 22). In addition, patients No. 3 and No. 6 showed decreased IgE binding to matrices generated with hesperidin, diosmin and the multistandard solutions. In general, however, the trend observed for lysozyme–polyphenol matrices was to show similar or higher IgE binding than native lysozyme. With regard to the matrices generated from ovalbumin and lemon peel extract, or the corresponding pure polyphenols identified as the main components, significant decreases in IgE binding relative to native protein controls were observed for hesperidin in three sera (from eight patients) and for rutin in four patients. In contrast, and as described for the assays performed with the polyphenols present in orange peel, patients No. 3, 5 and 6 showed the highest IgE binding to OVA–polyphenol matrices (Figure S23).

Even if only certain formulas are capable of inhibiting IgE binding in the case of lysozyme and OVA–polyphenol matrices, and responses vary from patient to patient, these results are collectively promising toward therapeutic approaches. The modulation of allergenic properties of known allergens is a trending strategy in immunotherapy [97]. In this context, some polyphenols from citrus extracts (or the synergistic effect of all antioxidants present in the extract) appear to be responsible for the low IgE binding observed. Differences between both allergens can be explained in terms of their physicochemical properties. whereas lysozyme is a small, globular protein whose epitopes are mostly exposed, and OVA is a big protein where the polyphenol binding can potentially expose hidden epitopes, which is consistent with the FTIR-spectral changes observed for the protein structure. OVA’s allergenicity is modulated through the digestion process, where the epitopes that are hidden will ultimately be exposed [98]. However, undigested OVA is believed to be the main initiator of the allergic response. Therefore, it would be an ideal candidate for oral immunotherapy in the cases of patients No. 1, 2, 4, 7 and 8, where the epitopes that these patients recognize are modified or hidden by the polyphenol interaction, either by direct binding to the epitope or indirectly by modifications of the tertiary structure of the protein. On the other hand, patients No. 3, 5 and 6, showed increased IgE binding, probably due to increased epitope exposure (FTIR revealed significant changes in the protein backbone after interaction with the polyphenols), although this effect could also be attributed to epitopes that would be exposed by OVA during gastrointestinal digestion.

Most lysozyme-pure polyphenols complexes show similar or higher IgE binding than that of native protein, although in principle this does not detract from interest. Oral immunotherapy treatments using low doses of the allergenic extracts or proteins are currently performed in clinical practice for hen’s egg allergy. In any case, there is always a risk of increasing adverse reactions instead of inducing tolerance, leaving aside that the failure rate in patients allergic to hen’s egg proteins is ca. 21% [99]. The addition of polyphenols, which often exhibit anti-inflammatory properties, to allergic proteins, represents a way of delivering such polyphenols to specific IgE-producing cells, thereby potentially impairing Th2-type inflammation and leading to the induction of allergen-specific tolerance [100]. Recent studies have shown that feeding polyphenol-rich vegetable extracts to OVA-sensitized mice reduced the expression of the GATA3 gene, which is a Th2 transcription factor, and increased the expression of TBX2, a Th1 transcription factor. This promotes the dominance of Th1 cells and, as a result, the improvement of the allergic condition. Furthermore, this diet maintained immune system homeostasis in mice by increasing the number of Treg cells and reducing the proliferation of Th17 ones [19]. Additional anti-inflammatory mechanisms accounting for allergen tolerance have focused on Foxp3 gene activation or interleukin (IL-10) production [101], which should be investigated in polyphenol–protein matrices to validate their potential in immunotherapy.

## 4. Conclusions

In this paper, we provide a proof of concept for allergen–polyphenol matrices that retain (and in the most favorable cases decrease) their hypoallergenic properties, being cheap and sustainable alternatives to traditional oral immunotherapy that involves the administration of reduced, yet hazardous, doses of neat food.

A series of hypoallergenic formulations merging polyphenols and two known hen’s egg protein allergens, namely lysozyme and ovalbumin, are reported and were tested in sensitized patients via indirect ELISA assays conducted on serum samples. The relationship between antioxidant activity and anti-allergenicity represents an open and controversial question without reaching clear-cut conclusions, especially when humans are considered, while animal models have proven some success. Probably, numerous mechanisms can operate at the molecular level. In any event, our work clearly demonstrated that citrus polyphenols, for which the antioxidant activity (radical scavenging) has been measured, can also induce decreased IgE-binding in polyphenol–protein matrices. Structural changes in native proteins, which may potentially account for hypoallergenic responses, have been monitored by infrared spectroscopy. In addition, our docking analyses simulating the interactions of some polyphenols (interacting strongly) at lysozyme’s binding site also delineate the major conformational changes of the secondary structure, depicting a challenging matter not previously considered. Yet, molecular dynamics simulations will be required in future studies to accurately show all of the stages of molecular interactions.

## Figures and Tables

**Table 1:** Identification of phenolic structures in orange peel extracts.

	Concentration Determined by HPLC (mg/g Extract)
Hesperidin	17.442 ± 0.039
Naringin	8.079 ± 0.029

**Table 2:** Identification of phenolic structures in lemon peel extracts.

	Concentration Determined by HPLC (mg/g Extract)
Hesperidin	9.662 ± 0.087
Eriocitrin	2.627 ± 0.071
Naringin	0.563 ± 0.038
Rutin	0.130 ± 0.023
Diosmin	0.115 ± 0.011

**Table 3:** Identification and quantitative analysis of phenolics from orange peel extract in the supernatant after matrix formation with lysozyme at room temperature *^a^*.

Polyphenol	Concentration in Extract (mg/g Extract) *^b^*	Concentration in Supernatant (mg/g Extract) *^b^*	% Interaction
Ratio 1:1			
Naringin	8.079 ± 0.029	4.676 *^c^* ± 0.008	42.1
Hesperidin	17.442 ± 0.039	8.756 *^c^* ± 0.018	49.8
Ratio 1:2			
Naringin	8.079 ± 0.029	5.936 *^c^* ± 0.065	26.5
Hesperidin	17.442 ± 0.039	11.024 *^c^* ± 0.024	36.8
Ratio 2:1			
Naringin	8.079 ± 0.029	3.093 *^c^* ± 0.011	61.7
Hesperidin	17.442 ± 0.039	4.673 *^c^* ± 0.017	73.2

*^a^* Determined by HPLC analyses. *^b^* Data expressed as mean ± standard deviation. *^c^* Significant differences (*p* < 0.05) relative to the initial concentration in the extract.

**Table 4:** Quantitative analysis of phenolics in supernatants after matrix formation *^a^*.

Polyphenol	Concentration in Starting Solution (mg/L) *^b^*		Concentration in Supernatant (mg/L) *^b^*	% Interaction
Naringin	36.04 ± 0.073		38.478 ± 0.054	-
Hesperidin	78.367 ± 0.478		18.745 *^c^* ± 0.020	76.1
Multistandard solution	Naringin	36.233 ± 0.597	38.517 ± 0.039	-
	Hesperidin	78.447 ± 0.048	15.525 *^c^* ± 0.027	80.2

*^a^* Determined by HPLC analyses. *^b^* Data expressed as mean ± standard deviation. *^c^* Significant differences (*p* < 0.05) relative to the initial concentrations.

**Table 5:** Quantitative analysis of phenolics (double concentration) in supernatants after matrix formation *^a^*.

Polyphenol	Concentration in Starting Solution (mg/L) *^b^*		Concentration in Supernatant (mg/L) *^b^*	% Interaction
Naringin	72.735 ± 0.353		70.412 *^c^* ± 0.014	3.2
Hesperidin	157.104 ± 0.006		23.899 *^c^* ± 0.019	84.8
Multistandard solution	Naringin	71.922 ± 0.079	66.521 *^c^* ± 0.042	7.5
	Hesperidin	156.011 ± 0.150	23.909 *^c^* ± 0.034	84.7

*^a^* Determined by HPLC analyses. *^b^* Data expressed as mean ± standard deviation. *^c^* Significant differences (*p* < 0.05) relative to the initial concentrations.

**Table 6:** Identification and quantitative analysis of phenolics from lemon peel extract in the supernatant after matrix formation with lysozyme at room temperature *^a^*.

Polyphenol	Concentration in Extract (mg/g Extract) *^b^*	Concentration in Supernatant (mg/g Extract) *^b^*	% Interaction
Ratio 1:1			
Naringin	0.563 ± 0.038	0.345 *^c^* ± 0.007	38.7
Hesperidin	9.662 ± 0.087	5.573 *^c^* ± 0.010	42.3
Eriocitrin	2.627 ± 0.071	1.532 *^c^* ± 0.010	41.7
Rutin	0.130 ± 0.023	0.067 *^c^* ± 0.004	48.5
Diosmin	0.115 ± 0.011	0.048 *^c^* ± 0.007	58.3
Ratio 1:2			
Naringin	0.563 ± 0.038	0.485 *^c^* ± 0.009	13.9
Hesperidin	9.662 ± 0.087	8.109 *^c^* ± 0.024	16.1
Eriocitrin	2.627 ± 0.071	2.175 *^c^* ± 0.063	17.2
Rutin	0.130 ± 0.023	0.099 ± 0.005	23.8
Diosmin	0.115 ± 0.011	0.078 *^c^* ± 0.009	32.2
Ratio 2:1			
Naringin	0.563 ± 0.038	0.232 *^c^* ± 0.003	58.8
Hesperidin	9.662 ± 0.087	3.279 *^c^* ± 0.019	66.1
Eriocitrin	2.627 ± 0.071	0.940 *^c^* ± 0.007	64.2
Rutin	0.130 ± 0.023	0.043 *^c^* ± 0.004	66.9
Diosmin	0.115 ± 0.011	0 *^c^*	100

*^a^* Determined by HPLC analyses. *^b^* Data expressed as mean ± standard deviation. *^c^* Significant differences (*p* < 0.05) relative to the initial concentration in the extract.

**Table 7:** Quantitative analysis of phenolics in supernatants after matrix formation *^a^*.

Polyphenol	Concentration in Starting Solution (mg/L) *^b^*		Concentration in Supernatant (mg/L) *^b^*	% Interaction
Naringin	2.502 ± 0.067		1.607 *^c^* ± 0.014	35.8
Hesperidin	43.852 ± 0.043		8.516 *^c^* ± 0.018	80.6
Diosmin	0.463 ± 0.006		0 *^c^*	100
Rutin	0.548 ± 0.017		0.072 *^c^* ± 0.002	86.9
Multistandard solution	Naringin	2.599 ± 0.081	2.986 ± 0.004	-
	Hesperidin	43.825 ± 0.034	2.494 *^c^* ± 0.010	94.3
	Diosmin	0.474 ± 0.012	0.037 *^c^* ± 0.003	92.2
	Rutin	0.570 ± 0.017	0.087 *^c^* ± 0.002	84.7

*^a^* Determined by HPLC analyses. *^b^* Data expressed as mean ± standard deviation. *^c^* Significant differences (*p* < 0.05) relative to the initial concentrations.

**Table 8:** Quantitative analysis of phenolics (double concentration) in supernatants after matrix formation *^a^*.

Polyphenol	Concentration in Starting Solution (mg/L) *^b^*		Concentration in Supernatant (mg/L) *^b^*	% Interaction
Naringin	5.159 ± 0.023		4.558 *^c^* ± 0.007	11.6
Hesperidin	87.910 ± 0.130		19.821 *^c^* ± 0.036	77.5
Diosmin	0.920 ± 0.010		0.031 *^c^* ± 0.003	96.6
Rutin	1.129 ± 0.028		0.120 *^c^* ± 0.005	89.4
Multistandard solution	Naringin	5.143 ± 0.018	5.844 ± 0.046	-
	Hesperidin	87.831 ± 0.028	3.930 *^c^* ± 0.016	95.5
	Diosmin	0.918 ± 0.012	0.045 *^c^* ± 0.002	95.1
	Rutin	1.135 ± 0.009	0.135 *^c^* ± 0.004	88.1

*^a^* Determined by HPLC analyses. *^b^* Data expressed as mean ± standard deviation. *^c^* Significant differences (*p* < 0.05) relative to the initial concentrations.

**Table 9:** Flavonoid derivatives reported in lysozyme docking calculations.

Flavonoid	ZINC ID	PDB	References
Daidzein	ZINC000018847034	2LYZ	[54]
Dihydromyricetin	ZINC000100037633	4D9Z	[88]
Diosmin	ZINC000004098512	2LYZ	[64]
(-)-Epicatechin	ZINC0000038703360	1JPO	[66]
(-)-Epigallocatechin gallate	ZINC000003870412	1JPO, 6LYZ	[66,89]
Fisetin	ZINC000000039111	6LYZ	[90]
Flavonol	ZINC000000057675	6LYZ	[91]
Genistein	ZINC000018825330	2LYZ	[54]
Hesperidin	ZINC000008143568	2WAR	[76]
7-Hydroxyflavone	ZINC000005934541	6LYZ	[91]
Kaempferol	ZINC000003869768	6LYZ	[92]
Luteolin	ZINC000018185774	6LYZ	[75]
Morin	ZINC000003881558	6LYZ	[90]
Myricetin	ZINC000003874317	4D9Z	[88]
Naringenin	ZINC000000156701	6LYZ	[93,94]
Naringin	ZINC000008143604	6LYZ	[93]
Quercetin	ZINC000003869685	1JPO	[55]
Pinostrobin	ZINC000000391894	6LYZ	[95]
Procyanidin B3	ZINC000042804873	6LYZ	[52]
Puerarin	ZINC000004098745	2LYZ	[54]
Rutin	ZINC000004096846	1HEW	[63]
Silybin	ZINC000002033589	2LYZ	[73]
α-Tocopherol	ZINC000004095858	6LYZ	[52]

**Table 10:** Crystal structures used in silico in the present work.

PDB ID	Resolution	pH	Co-Crystallized Ligand?
1DPX	1.65	4.5	
7BR5	1.00	4.5	(GlcNAc)_4_
4XAD	1.25	4.6	Galf-GlcNAc
6RT9	1.55	4.7	Sucrose
1HEW	1.75	4.7	(GlcNAc)_3_
6LYZ	2.00	7.4	
2LYZ	2.00	7.4	
1JPO	2.10	7.4	
2Z19	1.15	7.6	
4B4E	1.00	8.0	
5K2Q	1.10	8.5	
5K2P	1.24	8.5	
7JMU	1.20	8.0	
2FBB	1.46	8.4	

**Table 11:** Clinical features of patients allergic to hen’s egg.

Number of Patient	Age	Sex	Total IgE (kU/L)	Allergy Reaction	IgE to Hen’s Egg (kU/L)	IgE to Hen’s Egg Yolk (kU/L)	IgE to Hen’s Egg White (kU/L)	IgE to Gal d 2 (kU/L)	IgE to Gal d 4 (kU/L)
1	21	M	843	Anaphylaxis	26.9	17.6	36.1	18.8	39.2
2	19	M	43.7	OAS and AE with raw egg	0.9	0.41	0.92	0.24	NA
3	10	F	693	Syst.	5.25	3.25	6.2	4.13	0.19
4	38	M	55.5	Syst.	1.1	0.97	0.63	0.18	NA
5	63	F	15.6	Syst.	0.62	0.57	3.45	0.01	NA
6	22	F	192	Syst.	0.41	0.05	0.58	0.04	NA
7	11	M	2500	Syst.	6.8	3.15	4.1	5.03	NA
8	77	M	119	Syst.	3.05	0.48	3.46	3.61	NA

M: male, F: female, OAS: oral allergy syndrome, AE: angioedema, Syst.: systemic reaction, NA: not available.

## Data Availability

Data will be made available on request.

## Supplementary Materials

The following supporting information can be downloaded at: https://www.mdpi.com/article/10.3390/antiox13101154/s1: regression plots (Figures S1–S6); HPLC analyses showing the composition and concentration of polyphenols present in citrus peel extracts and polyphenol–protein matrices in the supernatants (Tables S1–S6); additional FT-IR spectra of native proteins and their modified secondary structure by non-covalent interactions with phenolics (Figures S7–S13);supplemental figures from the docking analyses with the corresponding self-explanatory legends for clarity (Figures S14–S21), and additional ELISA data (Figures S22–S23).
