# Supplementary material for: Antioxidant Activity and Hypoallergenicity of Egg Protein Matrices Containing Polyphenols from Citrus Waste
Source: Antioxidants (Basel). 2024 Sep 24;13(10):1154. doi: 10.3390/antiox13101154 (PMC11504875; doi:10.3390/antiox13101154)
Supplement: Supplementary file 1 [file antioxidants-13-01154-s001.zip › antioxidants-3169067-supplementary.pdf]

Antioxidant Activity and Hypoallergenicity of Egg Protein Matrices Containing Polyphenols from Citrus Waste

María Victoria Gil [1,*], Nuria Fernández-Rivera [1], Gloria Gutiérrez-Díaz [2], Jorge Parrón-Ballesteros [2], Carlos Pastor-Vargas [2], Diana Betancor [3], Carlos Nieto [4] and Pedro Cintas [1]

[1] Department of Organic and Inorganic Chemistry, IACYS-Green Chemistry and Sustainable Development Unit, Faculty of Sciences, University of Extremadura, 06006 Badajoz, Spain; nuriafr@unex.es (N.F.-R.); pecintas@unex.es (P.C.)

[2] Department of Biochemistry and Molecular Biology, Faculty of Chemistry, Complutense University of Madrid, 28040 Madrid, Spain; glogut01@ucm.es (G.G.-D.); jparron@ucm.es (J.P.-B.); cpasto01@ucm.es (C.P.-V.)

[3] Department of Allergy and Immunology, IIS-Fundación Jiménez Díaz, Universidad Autónoma de Madrid, 28049 Madrid, Spain; diana.betancor@quironsalud.es

[4] Department of Organic Chemistry, Faculty of Chemical Sciences, University of Salamanca, Pl. Caídos s/n, 37008 Salamanca, Spain; eneas@usal.es

* Correspondence: vgil@unex.es

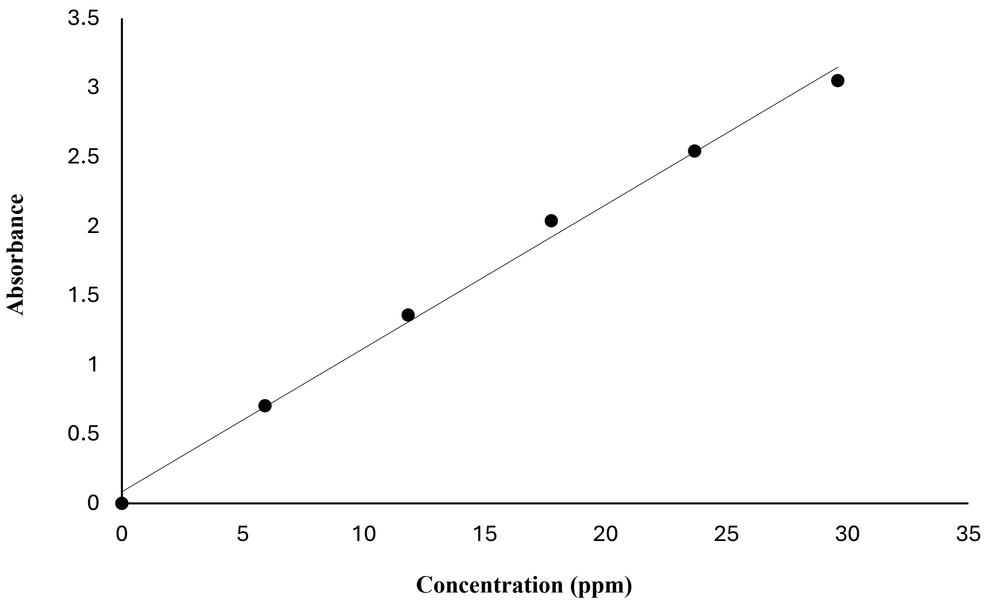

Figure S1. Linear relationship for determining the concentration of total polyphenolics in orange peels extracts.

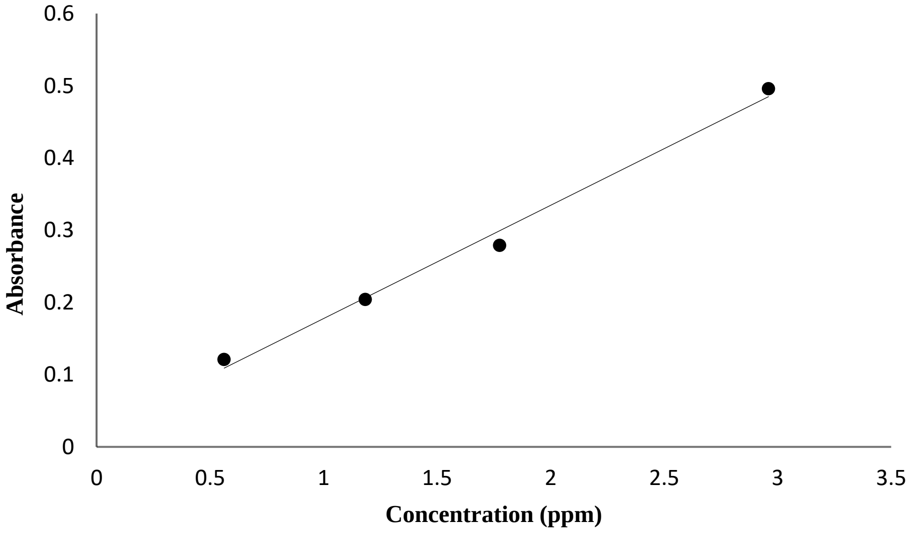

Figure S2. Linear regression for calculating the concentration of total polyphenolics in orange juice extracts.

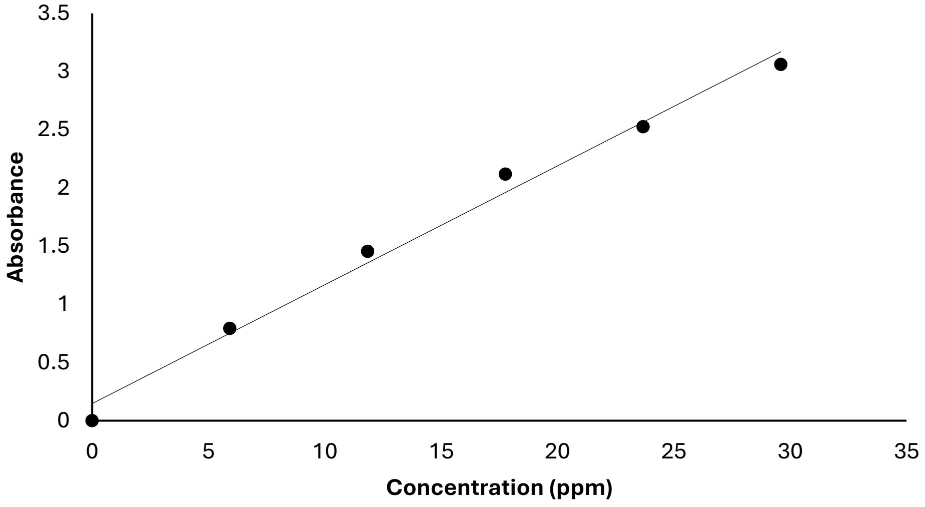

Figure S3. Linear relationship for determining the concentration of total polyphenolics in lemon peels extracts.

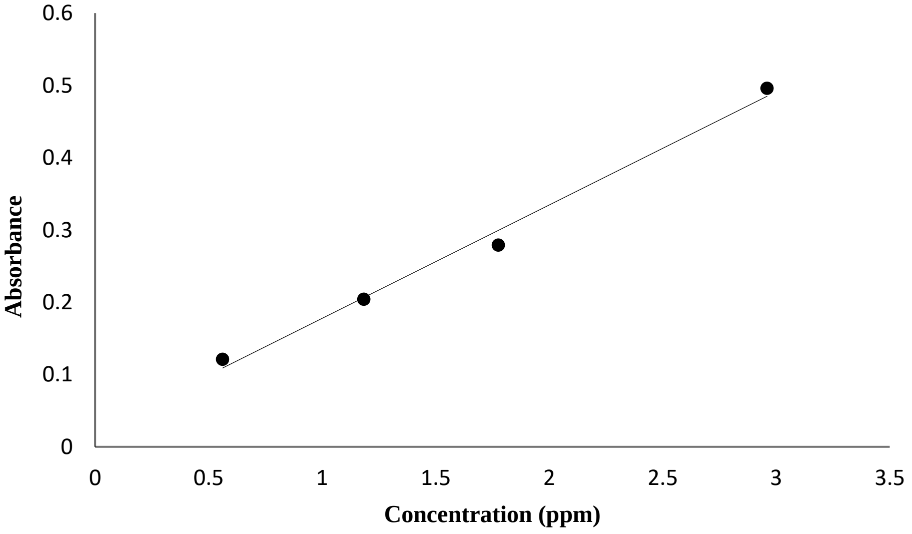

Figure S4. Linear regression for calculating the concentration of total polyphenolics in lemon juice extracts.

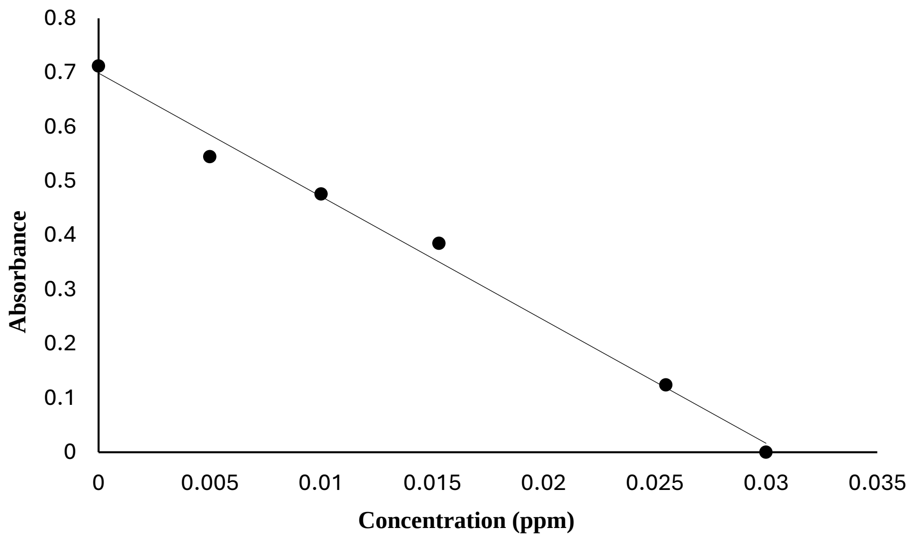

Figure S5. Linear plot obtained for antioxidant capacity reaction of orange peels extracts. The vitamin E analog Trolox was employed as external standard.

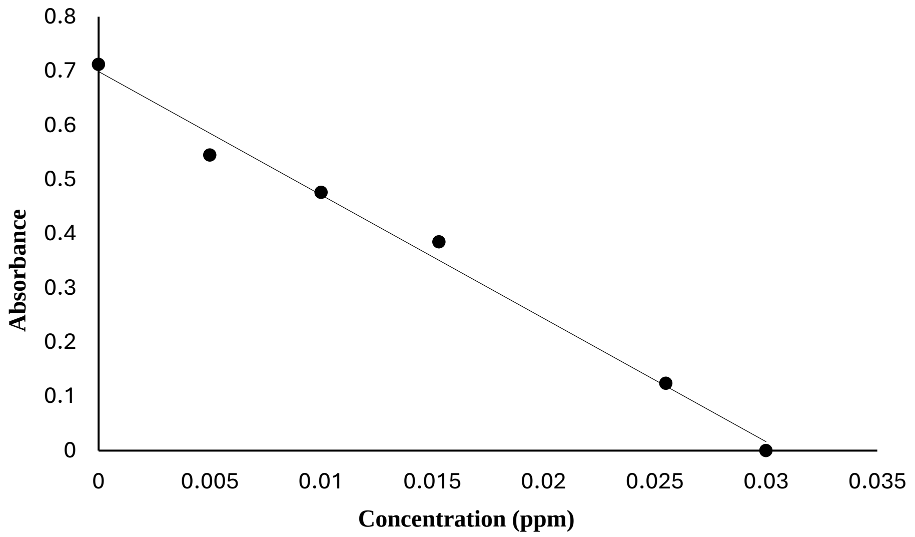

Figure S6. Linear plot obtained for antioxidant capacity reaction of lemon peels extracts. The vitamin E analog Trolox was employed as external standard.

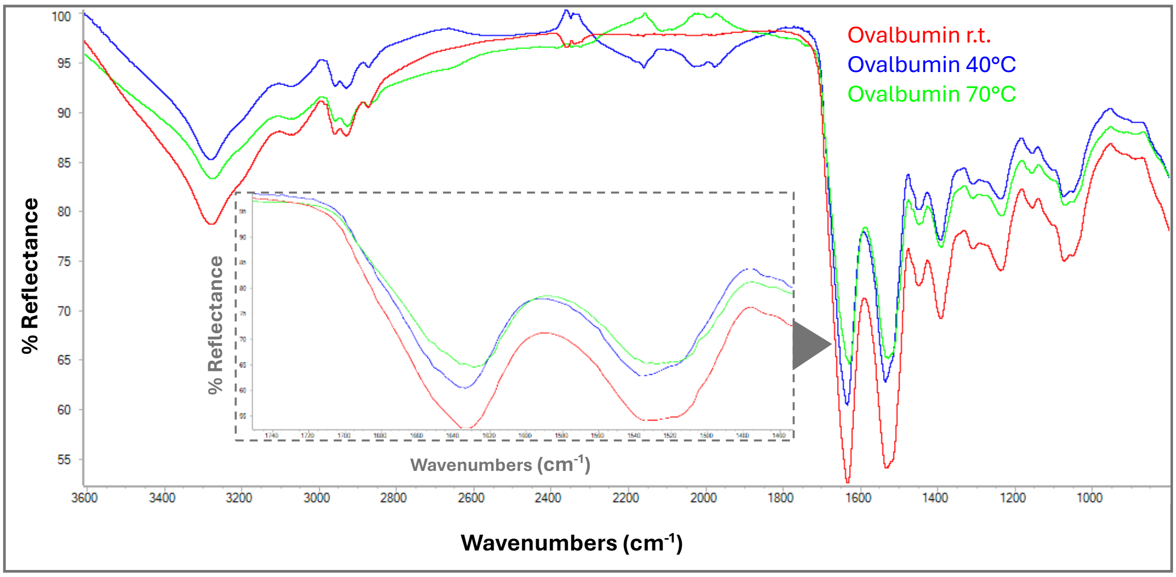

Figura S7. ATR-FTIR spectra recorded for ovalbumin at room temperature, 40 ºC, and 70 ºC. The inner inset shows an enlargement of the absorptions corresponding to amide I and II bands (1200-1800 cm^{-1}).

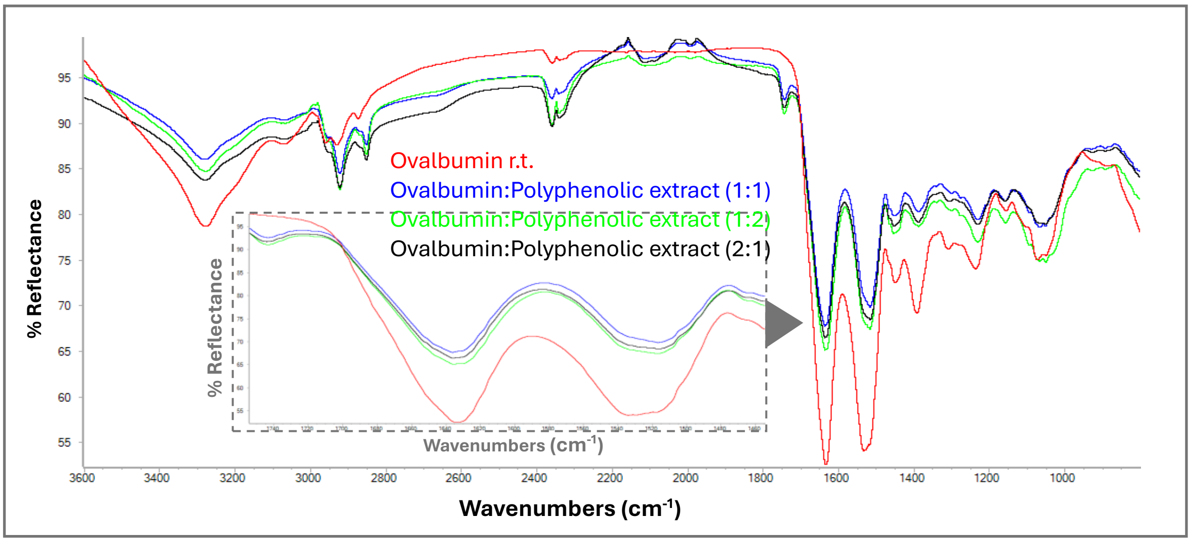

Figura S8. ATR-FTIR spectra of unmodified ovalbumin and its matrices with polyphenols from orange peels at room temperature. The inner inset shows an enlargement of the absorptions corresponding to amide I and II bands (1200-1800 cm^{-1}).

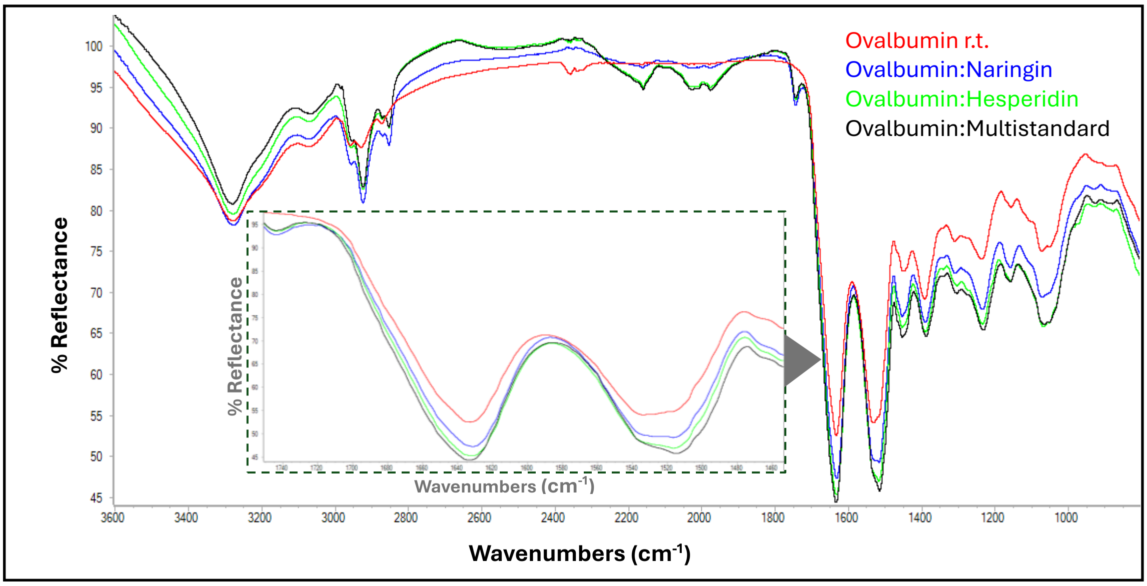

Figura S9. ATR-FTIR spectra of ovalbumin and its matrices with pure polyphenols and a multistandard solution. The inner inset shows an enlargement of the absorptions corresponding to amide I and II bands (1200-1800 cm^{-1}).

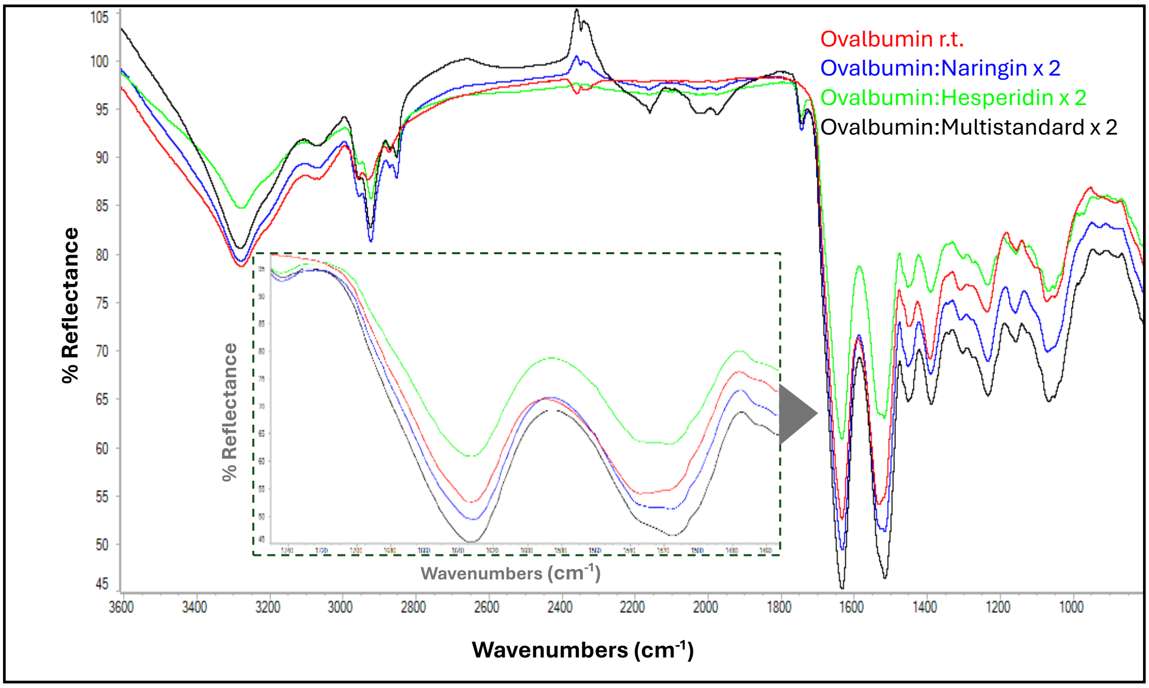

Figura S10. ATR-FTIR spectra of ovalbumin and its matrices with pure polyphenols and a multistandard solution with double concentrations of phenolics. The inner inset shows an enlargement of the absorptions corresponding to amide I and II bands (1200-1800 cm^{-1}).

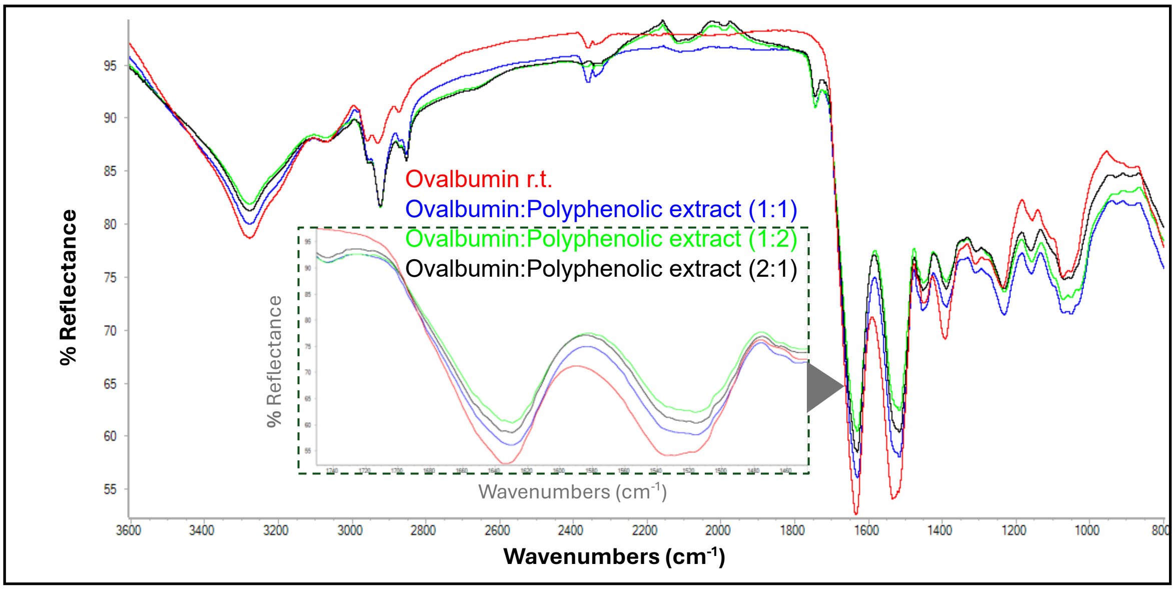

Figure S11. ATR-FTIR spectra of unmodified ovalbumin and its matrices with polyphenols from lemon peels at room temperature. The inner inset shows an enlargement of the absorptions corresponding to amide I and II bands (1200-1800 cm^{-1}).

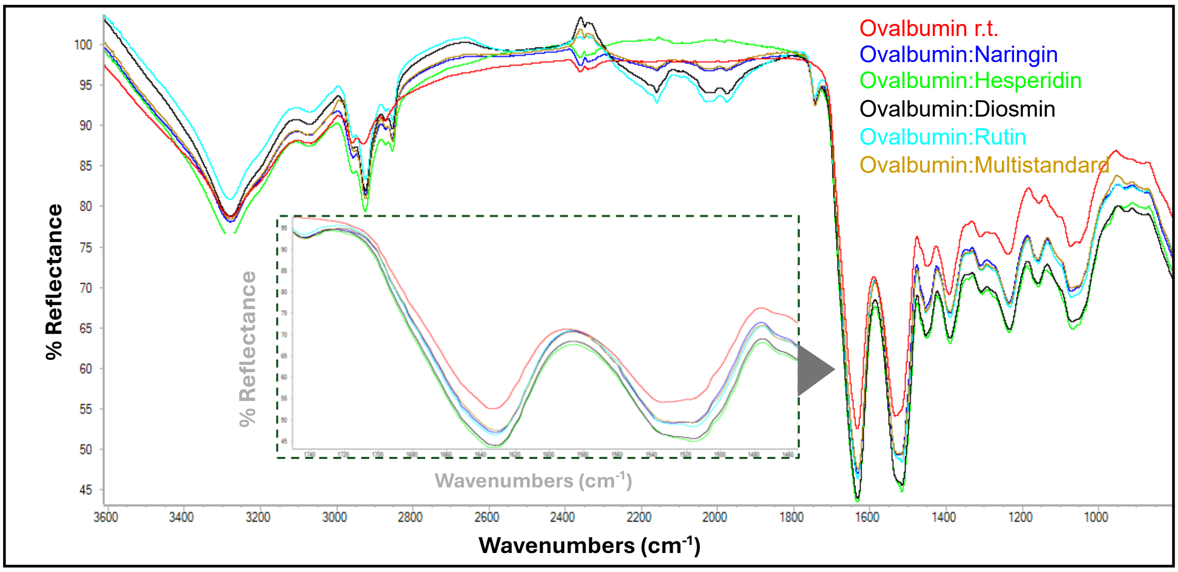

Figure S12. ATR-FTIR spectra of ovalbumin and its matrices with pure polyphenols and a multistandard solution. The inner inset shows an enlargement of the absorptions corresponding to amide I and II bands (1200-1800 cm^{-1}).

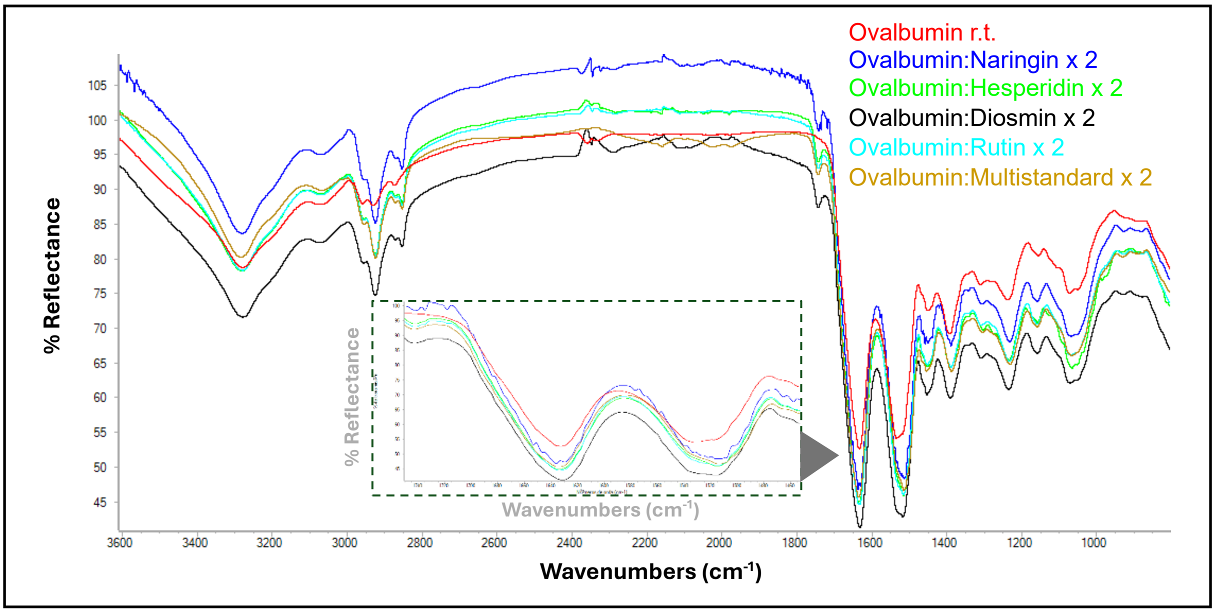

Figure S13. ATR-FTIR spectra of lysozyme and its matrices with pure polyphenols and a multistandard solution with double concentrations of phenolics. The inner inset shows an enlargement of the absorptions corresponding to amide I and II bands (1200-1800 cm^{-1}).

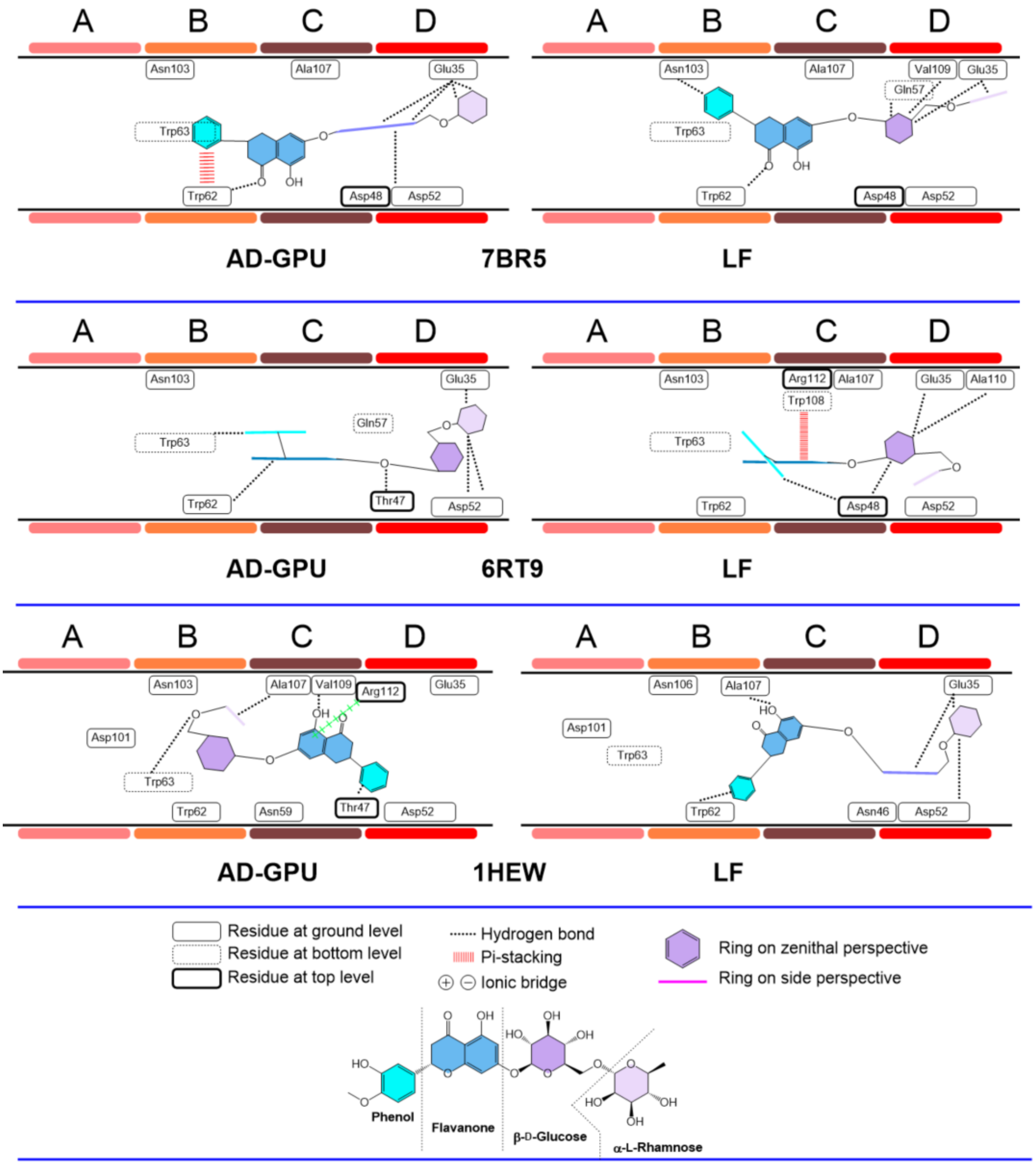

Figure S14. Hesperidin-lysozyme binding topologies (acidic pH) with 7BR5, 6RT9 and 1HEW, showing the relative position of the ligand to the ABCD pocket clefts.

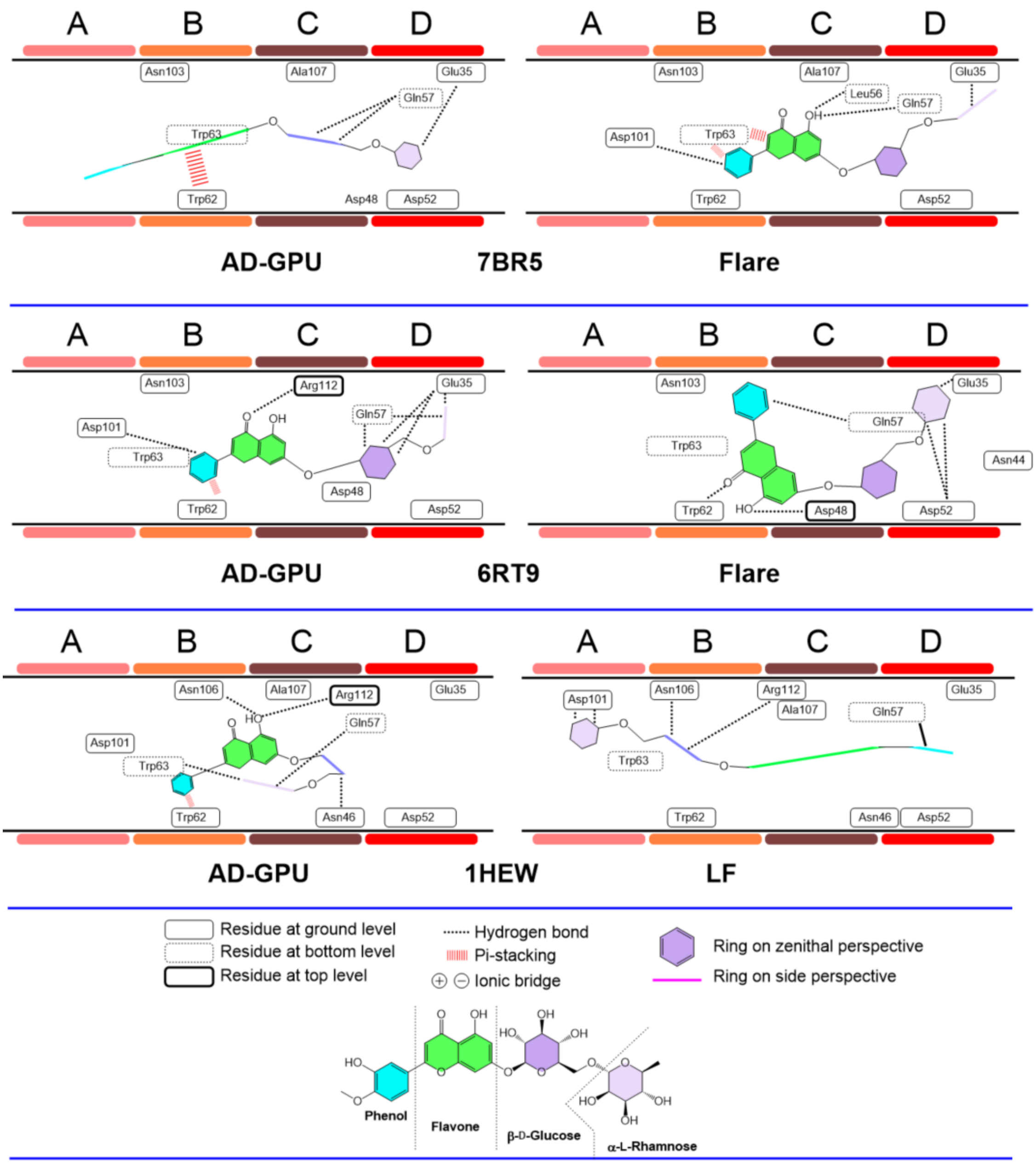

Figure S15. Diosmin-lysozyme binding topologies (acidic pH) with 7BR5, 6RT9 and 1HEW, showing the relative position of the ligand to the ABCD pocket clefts.

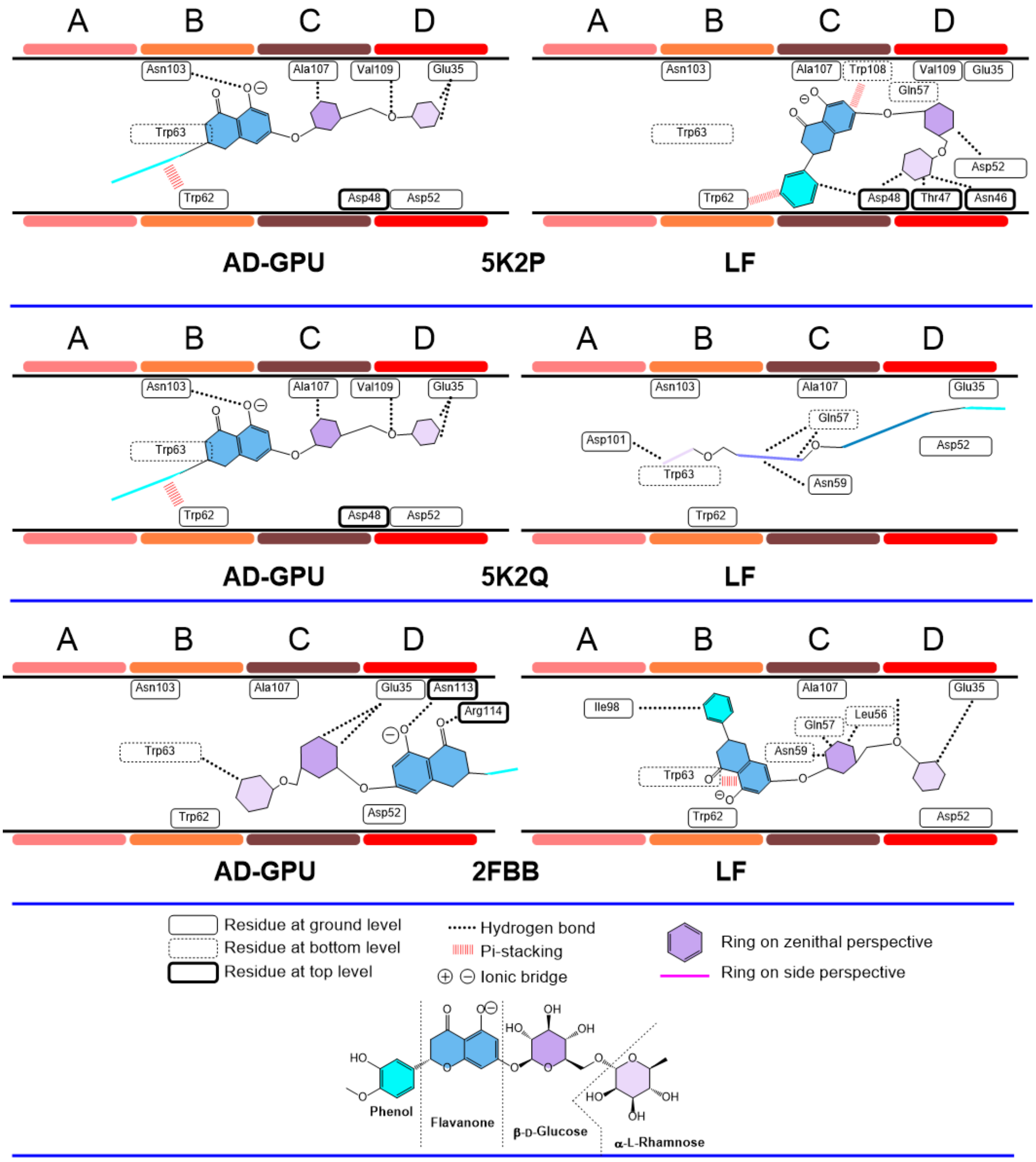

Figure S16. Hesperidin-lysozyme binding topologies (basic pH) with 5K2P, 5K2Q and 2FBB, showing the relative position of the ligand to the ABCD pocket clefts.

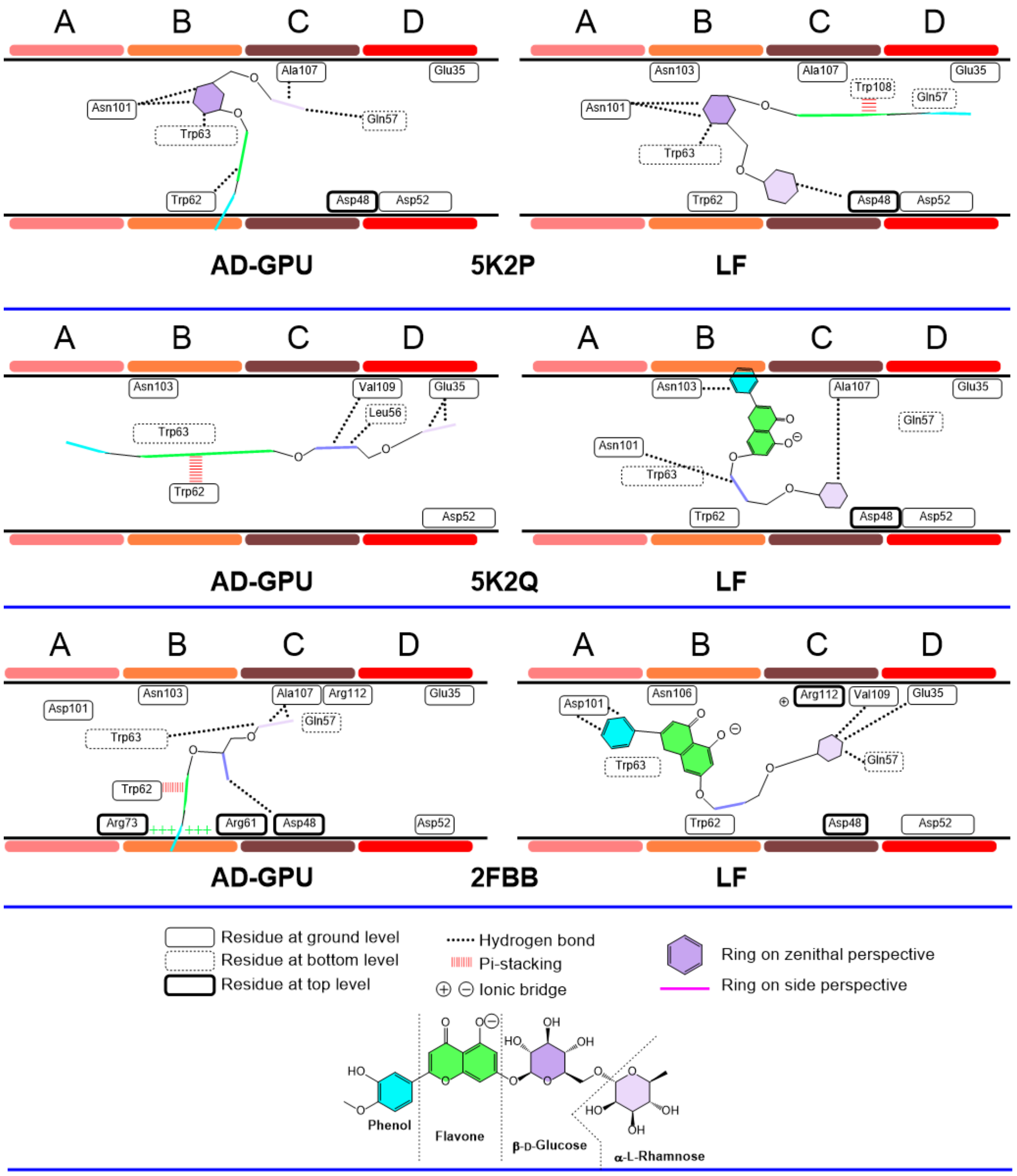

Figure S17. Diosmin-lysozyme binding topologies (basic pH) with 5K2P, 5K2Q and 2FBB, showing the relative position of the ligand to the ABCD pocket clefts.

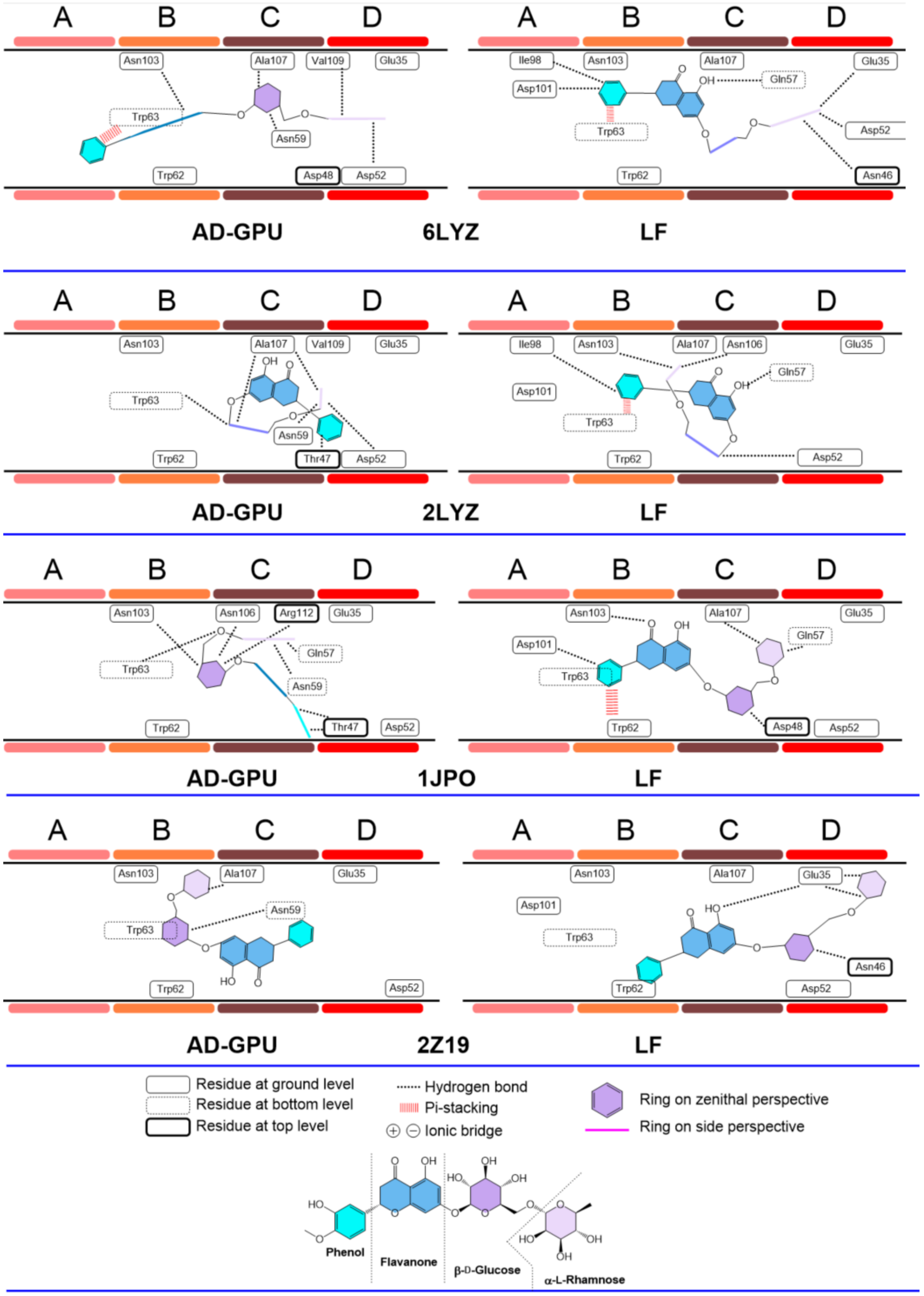

Figure S18. Hesperidin-lysozyme binding topologies (neutral pH, non-ionized ligand) with 2LYZ, 6LYZ, 1JPO and 2Z19, showing the relative position of the ligand to the ABCD pocket clefts.

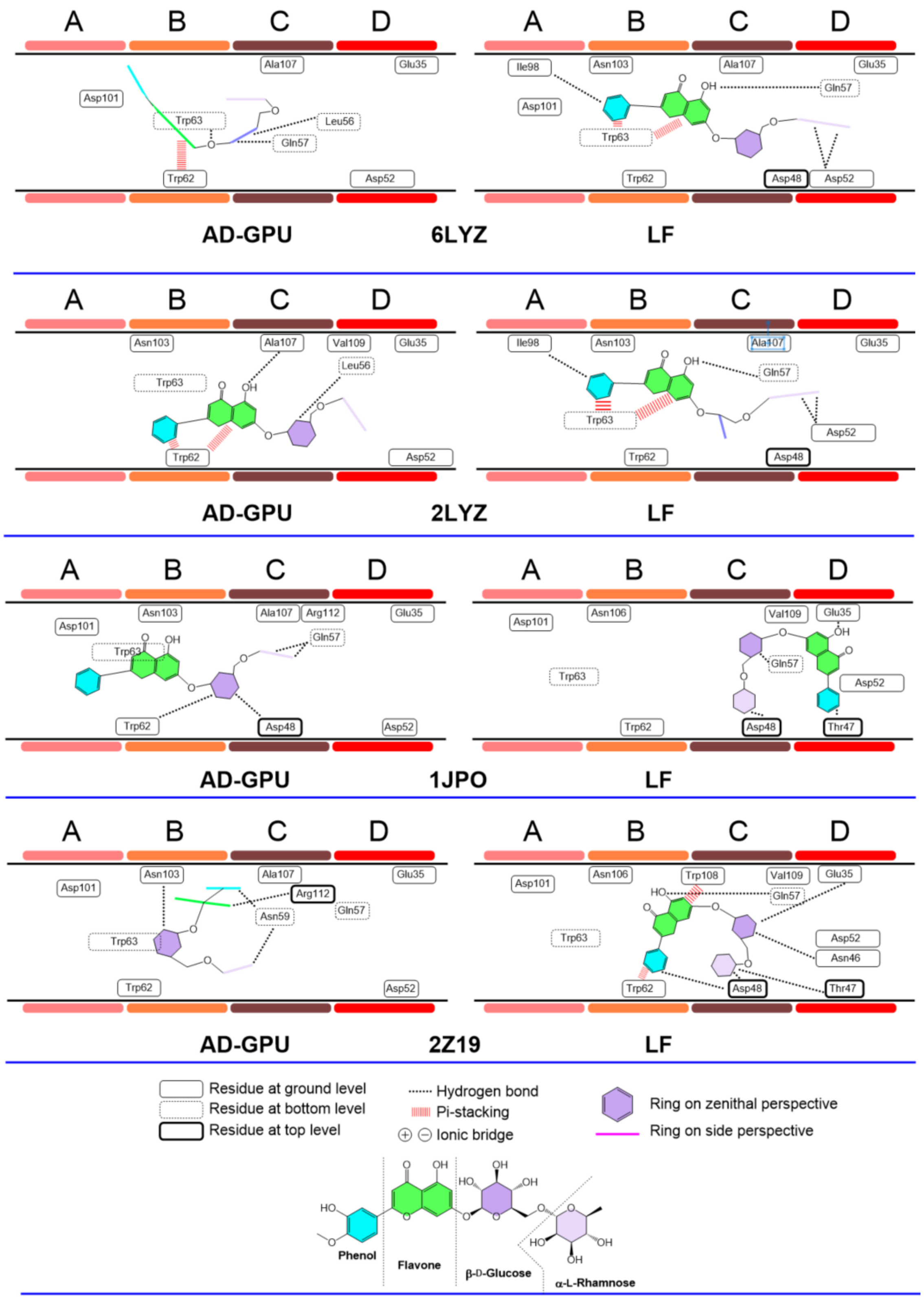

Figure S19. Diosmin-lysozyme binding topologies (neutral pH, non-ionized ligand) 2LYZ, 6LYZ, 1JPO and 2Z19, showing the relative position of the ligand to the ABCD pocket clefts.

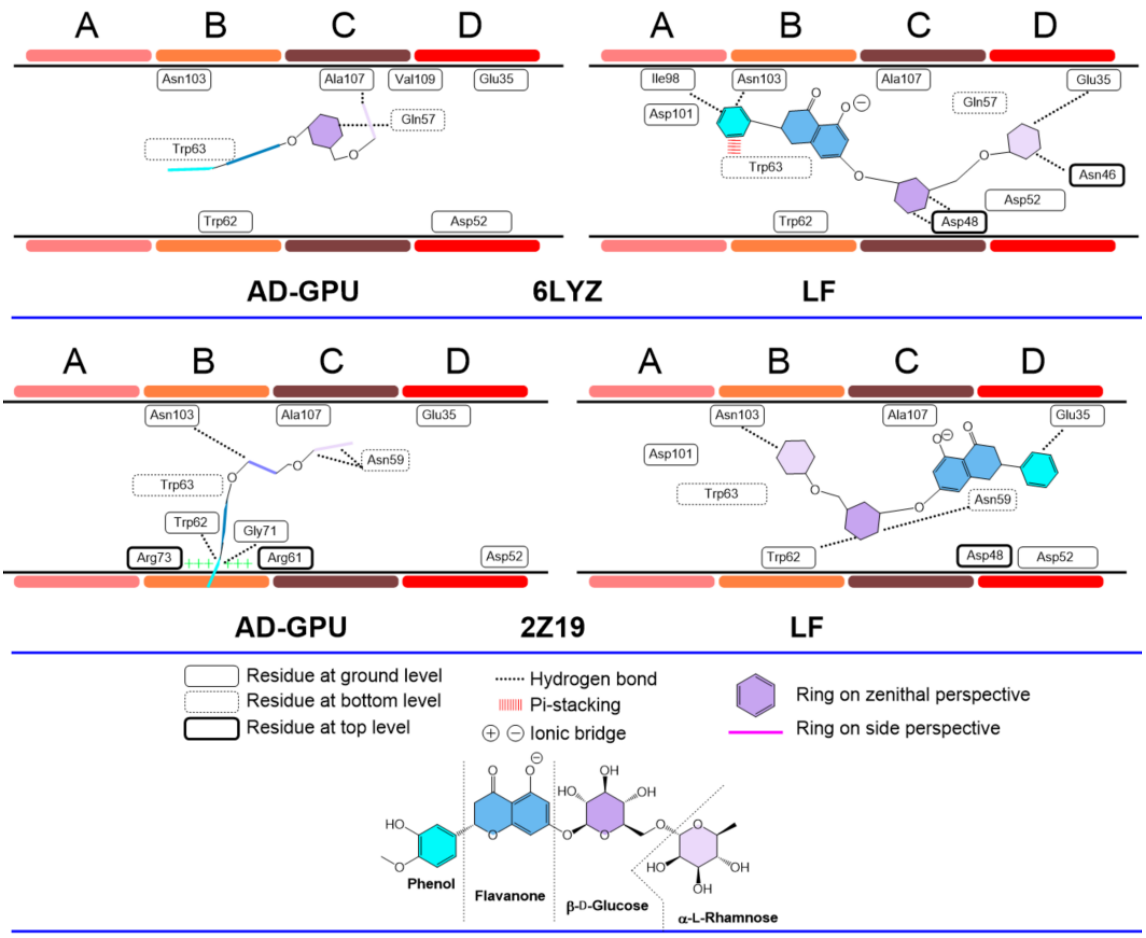

Figure S20. Hesperidin-lysozyme binding topologies (neutral pH, ionized ligand) with 6LYZ and Z19, showing the relative position of the ligand to the ABCD pocket clefts.

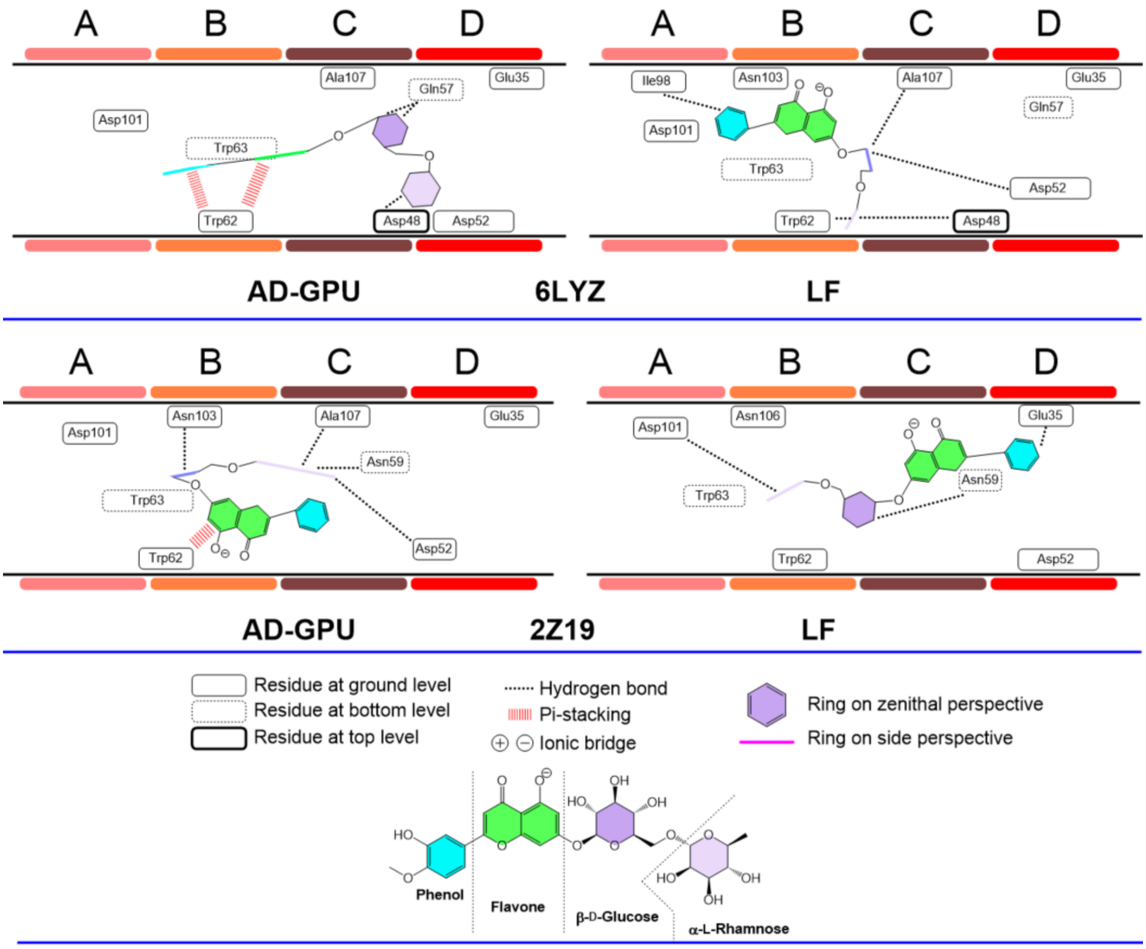

Figure S21. Diosmin-lysozyme binding topologies (neutral pH, non-ionized ligand) 6LYZ and 2Z19, showing the relative position of the ligand to the ABCD pocket clefts.

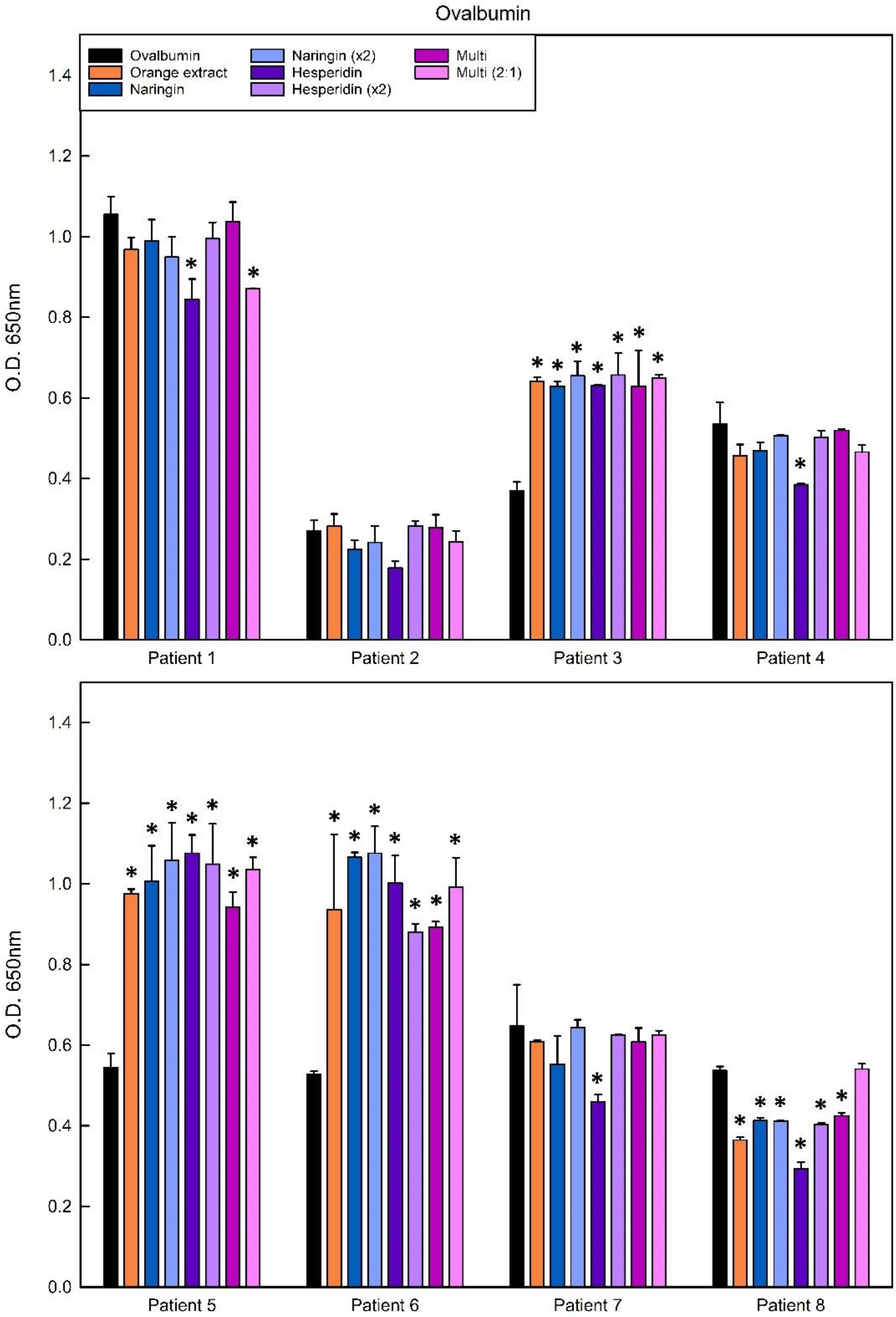

Figure S22. Values observed from indirect ELISA with diluted sera from eight patients allergic to hen egg ovalbumin, using the native protein as positive control and different protein-polyphenol present in orange peel matrices. Statistically significant differences between the matrices and the native protein were analysed by one-way ANOVA and are indicated by an asterisk ($p < 0.05$).

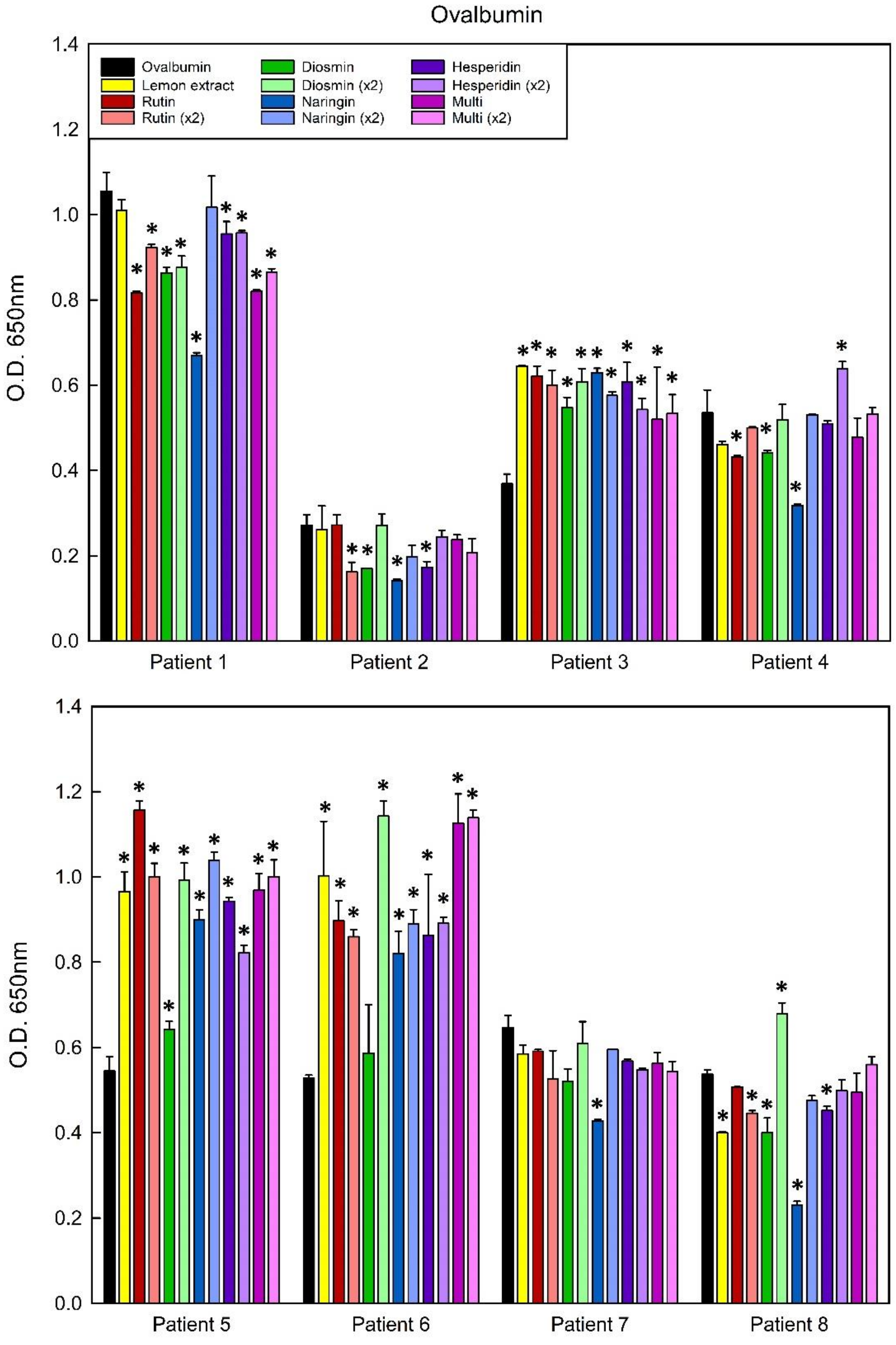

Figure S23. Resulting indirect ELISA plot with diluted sera from eight patients allergic to hen egg lysozyme, using the native protein as a positive control and different protein-polyphenol matrices from lemon peel. Statistically significant differences between the matrices and the native protein were analysed by one-way ANOVA and are indicated by an asterisk ($p < 0.005$).

Table S1. Identification and quantitative analysis of phenolics from orange peel extract in the supernatant after matrix formation with ovoalbumin at r.t.[a]

Polyphenol	Concentration in extract (mg/g extract)[b]	Concentration in supernatant (mg/g extract)[b]	% Interaction
		**Ratio 1:1**	
**Naringin**	8.079 ± 0.029	3.376[c] ± 0.018	58.2
**Hesperidin**	17.442 ± 0.039	6.668[c] ± 0.022	61.8
		**Ratio 1:2**	
**Naringin**	8.079 ± 0.029	5.084[c] ± 0.018	37.0
**Hesperidin**	17.442 ± 0.039	10.133[c] ± 0.006	41.9
		**Ratio 2:1**	
**Naringin**	8.079 ± 0.029	3.041[c] ± 0.019	62.4
**Hesperidin**	17.442 ± 0.039	6.114[c] ± 0.020	64.9

[a]Determined by HPLC analyses. [b]Data expressed as mean ± standard deviation. [c]Significant differences ($p < 0.05$) relative to the initial concentration in extract.

Table S2. Identification and quantitative analysis of phenolics from lemon peel extract in the supernatant after matrix formation with ovoalbumin at r.t.[a]

Polyphenol	Concentration in extract (mg/g extract)[b]	Concentration in supernatant (mg/g extract)[b]	% Interaction
		**Ratio 1:1**	
**Naringin**	0.563 ± 0.038	0.345[c] ± 0.044	38.7
**Hesperidin**	9.662 ± 0.087	5.561[c] ± 0.037	42.4
**Eriocitrin**	2.627 ± 0.071	1.487[c] ± 0.016	43.4
**Rutin**	0.130 ± 0.023	0.076[c] ± 0.007	41.5
**Diosmin**	0.115 ± 0.011	0.051[c] ± 0.002	55.6
		**Ratio 1:2**	
**Naringin**	0.563 ± 0.038	0.349[c] ± 0.017	38.0
**Hesperidin**	9.662 ± 0.087	5.826[c] ± 0.024	39.7
**Eriocitrin**	2.627 ± 0.071	1.557[c] ± 0.029	40.7
**Rutin**	0.130 ± 0.023	0.0727[c] ± 0.007	44.1
**Diosmin**	0.115 ± 0.011	0.058[c] ± 0.007	49.6
		**Ratio 2:1**	
**Naringin**	0.563 ± 0.038	0.265[c] ± 0.022	52.9
**Hesperidin**	9.662 ± 0.087	3.078[c] ± 0.037	68.1
**Eriocitrin**	2.627 ± 0.071	0.815[c] ± 0.012	69.0
**Rutin**	0.130 ± 0.023	0[c]	100
**Diosmin**	0.115 ± 0.011	0[c]	100

[a]Determined by HPLC analyses. [b]Data expressed as mean ± standard deviation. [c]Significant differences ($p < 0.05$) relative to the initial concentration in extract.

Table S3. Quantitative analysis of phenolics in supernatants after matrix formation.[a]

Polyphenol		Concentration in starting solution (mg/L)[b]	Concentration in the supernatant (mg/L)[b]	% Interaction
**Naringin**		36.04 ± 0.073	39.094 ± 0.011	-
**Hesperidin**		78.367 ± 0.478	13.930[c] ± 0.057	82.2
**Multistandard**	**Naringin**	36.233 ± 0.597	28.705[c] ± 0.018	20.8
**solution**	**Hesperidin**	78.447 ± 0.048	8.925[c] ± 0.074	88.6

[a]Determined by HPLC analyses. [b]Data expressed as mean ± standard deviation. [c]Significant differences ($p < 0.05$) relative to the initial concentrations.

Table S4. Quantitative analysis of phenolics (double concentration) in supernatants after matrix formation.[a]

Polyphenol		Concentration in starting solution (mg/L)[b]	Concentration in the supernatant (mg/L)[b]	% Interaction
**Naringin**		72.735 ± 0.352	96.372 ± 0.008	-
**Hesperidin**		157.104 ± 0.006	16.753[c] ± 0.026	89.3
**Multistandard**	**Naringin**	71.922 ± 0.080	70.119[c] ± 0.034	2.5
**solution**	**Hesperidin**	156.011 ± 0.150	13.659[c] ± 0.054	91.2

[a]Determined by HPLC analyses. [b]Data expressed as mean ± standard deviation. [c]Significant differences ($p < 0.05$) relative to the initial concentrations.

Table S5. Quantitative analysis of phenolics in supernatants after matrix formation.[a]

Polyphenol		Concentration in starting solution (mg/L)[b]	Concentration in the supernatant (mg/L)[b]	% Interaction
**Naringin**		2.502 ± 0.067	2.711 ± 0.022	-
**Hesperidin**		43.852 ± 0.043	4.754[c] ± 0.010	89.2
**Diosmin**		0.463 ± 0.006	0[c]	100
**Rutin**		0.548 ± 0.017	0.052[c] ± 0.005	90.5
	**Naringin**	2.599 ± 0.081	3.071[c] ± 0.003	-
**Multistandard**	**Hesperidin**	43.825 ± 0.034	1.748[c] ± 0.004	96.0
**solution**	**Diosmin**	0.474 ± 0.012	0[c]	87.8
	**Rutin**	0.570 ± 0.017	0.058[c] ± 0.002	100

[a]Determined by HPLC analyses. [b]Data expressed as mean ± standard deviation. [c]Significant differences ($p < 0.05$) relative to the initial concentrations.

Table S6. Quantitative analysis of phenolics (double concentration) in supernatants after matrix formation.[a]

Polyphenol		Concentration in starting solution (mg/L)[b]	Concentration in the supernatant (mg/L)[b]	% Interaction
**Naringin**		5.159 ± 0.023	5.167 ± 0.004	-
**Hesperidin**		87.910 ± 0.130	11.719[c] ± 0.026	86.7
**Diosmin**		0.920 ± 0.010	0.032[c] ± 0.002	88.5
**Rutin**		1.129 ± 0.028	0.142[c] ± 0.003	87.4
**Multistandard solution**	**Naringin**	5.143 ± 0.018	5.323 ± 0.006	-
	**Hesperidin**	87.831 ± 0.028	2.773[c] ± 0.021	96.8
	**Diosmin**	0.918 ± 0.012	0.042[c] ± 0.003	95.4
	**Rutin**	1.135 ± 0.009	0.113[c] ± 0.013	90.0

[a]Determined by HPLC analyses. [b]Data expressed as mean ± standard deviation. [c]Significant differences ($p < 0.05$) relative to the initial concentrations.
